# Mass Spectrometry-Based Investigation of Sugarcane Exposed to Five Different Pesticides

**DOI:** 10.3390/life13041034

**Published:** 2023-04-17

**Authors:** Thalisson A. de Souza, Gabriela C. S. Rodrigues, Pedro H. N. de Souza, Lucas S. Abreu, Laiane C. O. Pereira, Marcelo S. da Silva, Josean F. Tavares, Luciana Scotti, Marcus Tullius Scotti

**Affiliations:** 1 Multi-User Laboratory for Characterization and Analysis, Program of Natural and Synthetic Bioactive Products (PgPNSB), Health Sciences Center, Federal University of Paraíba, João Pessoa 58051-900, PB, Brazil; 2 Laboratory of Cheminformatics, Program of Natural and Synthetic Bioactive Products (PgPNSB), Health Sciences Center, Federal University of Paraíba, João Pessoa 58051-900, PB, Brazil; 3 Miriri Alimentos e Bioenergia S/A, Fazenda Miriri, Zona Rural, Santa Rita 58300-970, PB, Brazil; 4 Department of Organic Chemistry, Fluminense Federal University, Niteroi 24220-900, RJ, Brazil

**Keywords:** sugarcane, LC–MS, agrochemicals, nematodes, phenolic compounds

## Abstract

The use of agrochemicals has become a standard practice worldwide to ensure the productivity and quality of sugarcane crops. This study aimed to analyze the metabolic changes in sugarcane culms treated with five different nematicides. The experimental design was randomized in blocks, and agro-industrial and biometric variables were evaluated. The samples were extracted and then analyzed using LC–MS, LC–MS/MS, and LC–HRMS. The data obtained were submitted to statistical methods (PCA and PLS). Fragmentation patterns, retention time, and UV absorptions of the main features were analyzed. The plantations treated with carbosulfan (T4) obtained higher agricultural productivity and total recoverable sugar (TRS), while the use of benfuracarb (T3) was associated with lower growth and lower TRS. Statistical analysis revealed the contribution of the features at *m*/*z* 353 and *m*/*z* 515, assigned as chlorogenic acids, which discriminated the groups. The MS profile also supported the occurrence of flavonoids (C-glycosides and O-glycosides) in the samples.

## 1. Introduction

*Saccharum* spp. (sugarcane) is cultivated in more than 121 countries and represents the main crop used as a source of sugar and biofuel, supplying around 80% of sugar and 40% of bioethanol worldwide [1,2,3]. However, the production and quality of sugarcane are altered by several factors, including pests and pathogens, environmental conditions, and crop management [4].

Among the pests, nematodes are a major limitation to sugarcane production. There are many economically significant parasitic nematodes; however, three are widespread in Brazil and stand out due to the damage they cause to the crops: *Meloidogyne javanica*, *Meloidogyne incognita*, and *Pratylenchus zeae* [5,6]. The current literature details the extensive damage they cause to crops; the presence of high population densities of these species leads to yield losses that can reach up to 50% in the first cycle of the harvest [6,7].

In an attempt to control nematode infections, the use of agrochemicals has become an ongoing practice worldwide. There are eleven classes of nematicides at present, among them carbamates and sulfone derivatives have been applied for pest management in different crops [8,9]. The need for low-toxicity and environmentally friendly nematicides has guided the search for new alternatives. Natural products and biological control have also been considered to reduce phytoparasitic nematodes [10,11].

Plants respond to different abiotic and biotic stress conditions, specifically through the biosynthesis of secondary metabolites. Previous reports have described metabolic changes associated with sugarcane growth, response to predators and sugar content [12,13,14,15]. The application of omics techniques has allowed for a better understanding of the genome, physiology, and molecular biology of sugarcane [16,17]. Nevertheless, few studies have sought to investigate the effect of pesticides and other agrochemicals on sugarcane metabolism.

LC–MS analysis can be readily applied to predict the physical properties and physiological functions of sugarcane. Therefore, this work aims to verify the physiological response of sugarcane treated with five different nematicide controls (three synthetic compounds, one natural product, and one biological) using a metabolomics approach.

## 2. Materials and Methods

### 2.1. Plant Materials

The experiment was set up at Fazenda Santa Emília II, plot 14 (Latitude: 6°49′52.88″ S, longitude: 34°58′01.18″ W) at Miriri Alimentos e Bioenergia S/A during the summer planting of the agricultural year 2018/2019. Miriri Alimentos e Bioenergia S/A is located in the rural area of the municipality of Santa Rita-PB, on the banks of the BR-101 (7°6′59″ S, 34°58′52″ W), 43 km away from the capital, João Pessoa, Brazil.

**Figure 1:**
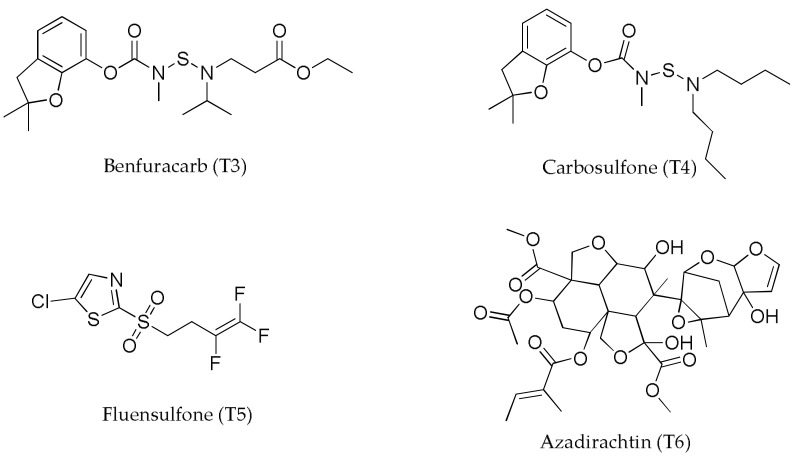
Chemical structure of nematicides.

For the implementation of the experiment, a randomized block design with five replications was used (Supplementary Information) using five nematicides as treatments: one biological product [*Bacillus subtilis* and *Bacillus licheniformis* (T2)], three with chemical actives (benfuracarb (T3), carbosulfan (T4), fluensulfone (T5)) and one natural product (azadiracthin (T6)), Figure 1. Treatment 1 (T1) was used as control and was not subjected to any nematicide. The amounts supplied for each treatment varied following the manufacturer’s recommendations; 0.20 kg ha^−1^ for T2, 5 L ha^−1^, 4 L ha^−1^, 1 L ha^−1^, and 2 L ha^−1^ were added for T3 to T6, respectively. The presence of *Meloidogyne* and *Pratylenchusin* species in the fields was verified before plot randomization.

Samples of sugarcane seedlings (Variety: RB92579) including all plant material (leaf, culms, and root) were randomly collected between 8:30 and 9:00 a.m. 30, 65, 95, and 125 days after planting in plots 01, 03, and 05. The plant material was dried in an oven (40 °C) for 48 h, ground in a knife mill, and stored in a freezer at −20 °C.

### 2.2. Preparation of Extracts and Fractions

The samples were obtained from 200 mg of milled sugarcane culms extracted in H_2_O/EtOH/IPA in a ratio of 30:45:25 (*v*/*v*), followed by sonication for 15 min. All extracts were prepared in triplicate. Next, the samples were dried in a reduced pressure system, resuspended in MeOH/H_2_O in a ratio of 1:1 (*v*/*v*), and cleaned up in C_18_ cartridges eluted with MeOH. After that, an aliquot of each fraction was collected and diluted for LC–MS^n^ analysis [18].

### 2.3. Liquid Chromatography–Mass Spectrometry Instrumentation, Conditions, and Compound Identification

A Shimadzu (Kyoto, Japan) high-performance liquid chromatography (HPLC) system was used, composed of an LC-20AD solvent pump unit (flow rate of 600 μL/min), a DGU-20A5 online degasser, a CBM-20A system controller, and an SPD-M20A (190–800 nm) diode array detector (DAD). Injections (20 μL) were performed using an autosampler (model SIL-10AF). The LC separation was performed on a Kromasil C18 5 μm 100 Å, 250 × 4.6 mm (Kromasil, Bohus, Sweden) analytical column. The mobile phase consisted of 0.1% formic acid in water (solvent A) and methanol (solvent B). An exploratory linear gradient (5 to 100% solvent B) was performed for an elution time of 40 min.

An HPLC Shimadzu (Kyoto, Japan) coupled with an amaZon X (Bruker Daltonics, Billerica, MA, USA) with an electrospray ion (ESI) source, was used to perform ESI-MSn. The analysis parameters were as follows: capillary 4.5 kV, ESI in negative mode, final plate offset 500 V, 40 psi nebulizer, dry gas (N2) with a flow rate of 8 mL/min and a temperature of 200 °C. Collision-induced dissociation (CID) fragmentation was achieved in the amaZon X in auto-MS/MS mode using the enhanced resolution mode. The mass spectra (*m*/*z* 50–1000) were recorded every 2 s. These samples were then injected again into an HPLC system coupled to a micrOTOF II mass spectrometer (Bruker Daltonics, Billerica, MA, USA) for high-resolution electrospray ionization mass spectrometry (HR-ESI-MS) analyses using the same method as previously reported [19].

Based on UV data collected using DAD, fragmentation patterns and the molecular formulas obtained from ion-trap (MS^2^ and MS^3^) and HRMS experiments [20], the main compounds present in sugarcane culm extracts were annotated.

### 2.4. Statistical Analysis (PCA and PLS)

The LC–MS spectral files were converted to mzXML format. The data were preprocessed (feature detection, sample alignment, and peak correspondence) in XCMS Online [21]. The integral values of each feature (*m*/*z*) were analyzed as a function of retention time. After that, the files were converted to CSV format. Principal component analysis (PCA) and partial least squares discriminant analysis (PLS-DA) of the data was performed in Unscrambler, version 9.7 (CAMO Process AS, Oslo, Norway). Area normalization was applied to each sample. “Tons of sugar” was used as the category variable in PLS-DA.

### 2.5. Biometric Variables

The biometric variables of plant height (PH), stem diameter (SD), and the number of plants per linear meter (NP) were measured following the Kuijper methodology [22]. The PH was measured using a measuring tape graduated in centimeters measured from the base of the plant to the first visible dewlap (that is, leaf insertion +1). The SD was obtained from the median region of the culms with the aid of a digital caliper with a precision of 1 mm. The NP was obtained by counting the number of stems in the central row divided by the furrow length.

### 2.6. Agro-Industrial Variables

Agricultural productivity was estimated, and the industrial performance of the treatments was analyzed after harvesting. The following agro-industrial variables were evaluated: agricultural productivity (t·ha^−1^), total recoverable sugar (TRS), and total sugar per hectare (TSA). The agricultural productivity was calculated by transforming the plot weight into kg using the following equation: Total plot weight × 10/plot area in m^2^. The TRS from the samples in each treatment was determined in the sucrose laboratory of Miriri Alimentos e Bioenergia S/A, applying the method reported to the São Paulo State Council of Sugarcane, Sugar, and Alcohol Producers [23]. The TSH was calculated using the expression: agricultural productivity × TRS/1000.

## 3. Results

### 3.1. Agro-Industrial and Biometric Variables

Initially, the values of the agro-industrial and biometric variables provided data on agricultural productivity and the effects of each treatment on sugarcane crops.

The biometric characteristics of sugarcane were recorded (Table 1). Plant height (PH) was stable during the cutting period, the general average height corresponded to 2.42 m, and lower growth was observed in treatment 3 (benfuracarb) with an average height of 2.24 m. In addition, a lower number of plants per meter was observed under this treatment. In general, the stem growth was similar for all five treatments, with the plants exposed to treatment 2 (*Bacillus subtilis* and *Bacillus licheniformis*) showing a greater height of 2.52 m, followed by those exposed to treatment 4 (carbosulfan). The stem diameter developed consistently in the treatments.

The current study shows that certain nematicides interfere in sugarcane crop productivity, demonstrated by TSH (tons of sugar per hectare). Treatment 4 (carbosulfan) had the best performance for TSH (13.56 t·ha^−1^), whereas treatment 3 (benfuracarb) obtained the lowest yield (11.87 t·ha^−1^). To calculate the TSH variable, the TRS (total recoverable sugar) of each treatment was used. The TRS is an indicator that represents the sugar contained in sugarcane and is used to quantify sugar and ethanol production, as well as indicate cane quality. In Brazil, producers are currently remunerated based on the TRS content that the raw material presents after it is harvested [24]. A detailed analysis of TRS by treatment and block can be seen in the Supplementary Information (Table S1).

Regarding the yield measured by the weight (kg), treatment 3 (benfuracarb) had the lowest value, 506 kg, while sugarcane from treatment 4 (carbosulfan) had the highest weight, 574 kg. When the number of plants per meter was evaluated, treatment 5 (fluensulfone) presented the best result. Although treatment 2 produced the tallest plants, the plants with the best development were exposed to treatment 4 (carbosulfan), which obtained the best results for t·ha^−1^, TRS, and TSH. In addition, the growth of sugarcane exposed to treatment 3 was affected, and the resulting plants were smaller than the control.

### 3.2. Statistical and Metabolic Profile

After analyzing the agro-industrial and biometric variables, the metabolome of sugarcane culms treated with different nematicides was analyzed in order to evaluate the biochemical effects of the changes previously observed.

Mass spectrometry analysis indicated the presence of common features at *m*/*z* 341, *m*/*z* 683, *m*/*z* 377, *m*/*z* 439, and *m*/*z* 533 in the six treatments (Supplementary Information). Sucrose was the main sugar identified based on fragmentation patterns. The deprotonated form and adducts of sucrose were also detected at *m*/*z* 341 [M−H]^−^, *m*/*z* 377 of chlorine [C_12_H_22_O_11_+Cl]^−^, *m*/*z* 439 of phosphate [M+H_2_PO_4_]^−^, and *m*/*z* 533 of citric acid [C_18_H_30_O_18_−H]^−^ [25,26].

**Figure 2:**
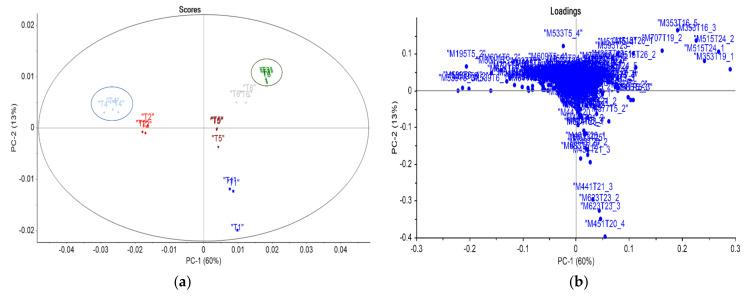
Graph of PCA scores (**a**) and loadings (**b**) (PC1 vs. PC2) for treatments 1 to 6 of collection 4 analyzed using mass spectrometry: treatment 1 (navy blue), treatment 2 (red), treatment 3 (green), and treatment 4 (blue), treatment 5 (brown), and treatment 6 (gray).

PCA analyses (Figure 2) revealed a particular discrimination pattern between treatments 1 to 6. The score plots, PC1 X PC2 explain 73.0% of the total variance of the original data. The results showed good discrimination of treatments 3 (benfuracarb) and 4 (carbosulfan) among other groups. The loadings plot shows the contribution of many features identified after an in-depth investigation using mass spectrometry analysis.

**Figure 3:**
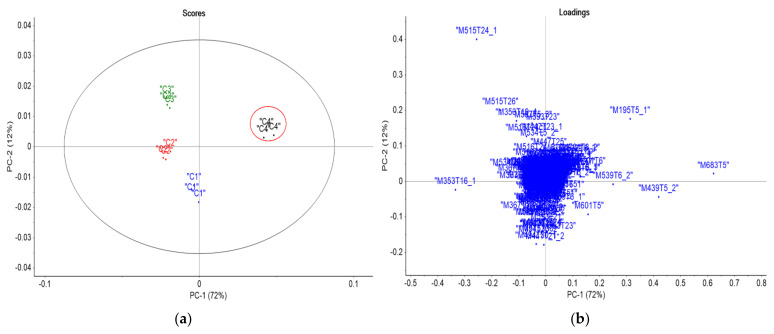
Graph of PCA scores (**a**) and loadings (**b**) (PC1 vs. PC2) for treatment 4 analyzed using mass spectrometry: collection 1 (blue), collection 2 (red), collection 3 (green), and collection 4 (black).

For treatment 4 (Figure 3), PCA analysis showed metabolomic differences between the collections conducted over time. PC1 and PC2 explained 84.0% of the total variance for this treatment. The scores graph showed four sample cluster regions. The loadings graph showed the contribution of features at *m*/*z* 353 and 515 [M−H]^−^ for collections 2 and 3, whereas the features at *m*/*z* 683[2M−H]^−^ and *m*/*z* 439 contributed to differentiate collection 4. These findings indicated metabolic changes over time, especially between collections 3 and 4.

**Figure 4:**
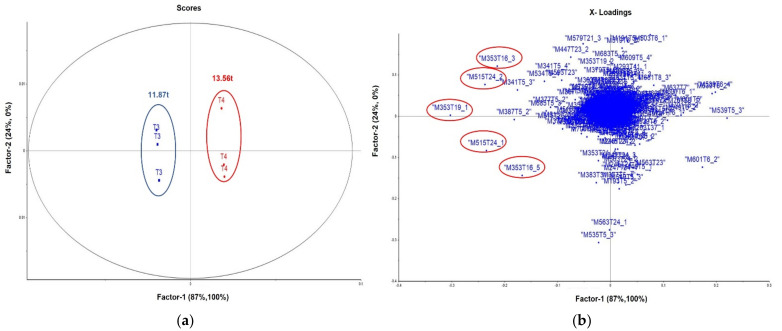
Graph of scores (**a**) and loadings (**b**) of PLS-DA (LV 1 vs. LV 2) with treatments 3 (blue) and 4 (red) analyzed by mass peaks of the compounds identified in the samples.

In order to identify which features contributed to discriminate the groups and analyze their possible relation with the higher sugar productivity in treatment 4, a partial least squares discriminant analysis (PLS-DA) comparing treatments 3 and 4 (collection 4) was performed. The coefficient of determination (r2) and coefficient of internal prediction (q2—leave-one-out-cross-validation) are 0.998 and 0.995, respectively. The analysis of the loading graphs for these groups indicated that LV (leverage) 1 and LV 2 explained 87% of the total variance in the original data (Figure 4). The score graph showed a good grouping of treatments 3 and 4, and the loadings revealed the contribution of *m*/*z* 353 and *m*/*z* 515.

Comparing treatments 2 and 4 with the control group (treatment 1), PC1 and PC2 explained 83.0% of the total variance. The scores graph showed three sample cluster regions. In treatment 1, the main features that contributed to discrimination in the loading graph were *m*/*z* 623 *m*/*z* 441, *m*/*z* 451, *m*/*z* 353, and *m*/*z* 515 (Figure 4). Treatments 2 and 4 differ on *m*/*z* 195, *m*/*z* 535, and saccharose adducts, as previously discussed (Supplementary Information). Treatments 3 and 6 were also evaluated using the same approach (Supplementary Information). The scores and loadings plots revealed a similar profile; the features at *m*/*z* 353, *m*/*z* 515 and their isomers were associated with treatment 3 (Supplementary Information).

In view of the recurrent presence of some features, a targeted analysis was conducted to annotate the compounds and analyze their possible relation to sugar productivity. As a result, 27 substances were annotated in the sugarcane culm extracts. For the evaluation of samples, data obtained from the literature were employed. The retention times, mass spectral data, and peak assignments for the compounds identified using negative ionization are described in Table 2.

The profile of secondary metabolites shows the presence of phenolic compounds. Regarding flavonoids, seventeen C-glycosides were identified by comparing their MS/MS spectra with those available in the literature. The ion fragments [M−H−60]^−^, [M−H−90]^−^, and [M−H−120]^−^ are the characteristic diagnostic ions related to the [(O-C1 and C2–C3)], [(O-C1 and C3–C4)], and [(O-C1 and C4–C5)] cross-ring cleavages of the sugar units, respectively [33]. Furthermore, it was also possible to identify the ions [aglycone + (41 or 71)]^−^ and [aglycone + (83 or 113)]^−^ for mono-C and di-C-glycosides, respectively. These fragment ions represent aglycone plus the sugar residues that have remained bound to them, which enabled the identification of the aglycones apigenin, luteolin, and diosmetin [34]. Fifteen of seventeen flavonoid-C-glycosides in sugarcane have been described previously [27].

Moreover, tree phenolic acid and tree flavonoid-O-glycosides, such as luteolin-7-O-glucoside, tricin-7-O-α-L-rhamnosyl-glucuronide, and tricin-7-O-glucuronide-sulfate, were identified based on their fragmentation pathways and literature data [28,32]. The main features *m*/*z* 353 and *m*/*z* 515 were detected and assigned to chlorogenic acids (CQA). For the identification and characterization of CQA, four main ion fragments were observed: Q1 [quinic acid−H]^−^, C1 [caffeic acid−H]^−^, Q_2_ [quinic acid−H_2_O−H]^−^, and C2 [caffeic acid−CO_2_−H] [35].

**Figure 5:**
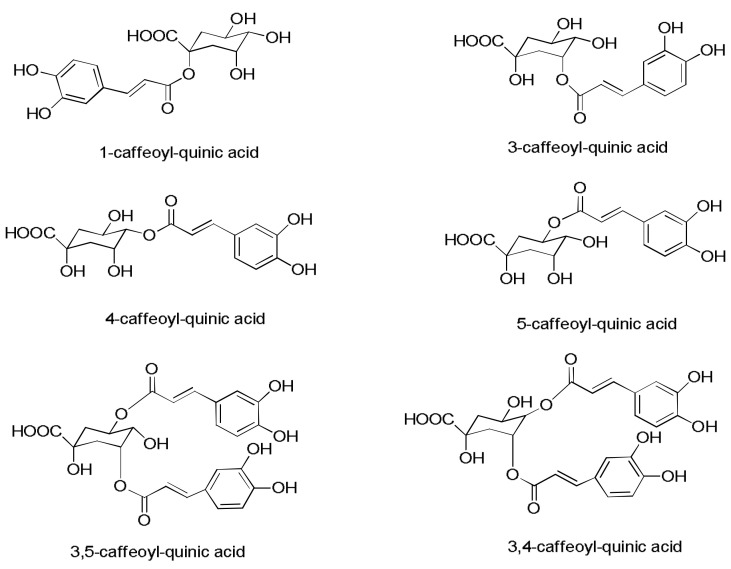
Structures of the main features at *m*/*z* 353 and *m*/*z* 515 identified as chlorogenic acids.

Four peaks with *m*/*z* 353, representative of the four isomers of CQA, were detected in the extracts. The differentiation of the isomers was possible with the analysis of the intensity of the ion fragments based on literature data [36]. In addition, one coumaroylquinic acid with *m*/*z* 337 possessing similar ion fragments has been identified [29]. Two di-caffeoylquinic acid isomers with precursor ions *m*/*z* 515 and ion fragments with *m*/*z* 353 ([CQA−H]^−^, Q1, C1, Q2, and C2) were also observed. The CQAs identified are displayed in Figure 5. Similar to CQA, the isomers were distinguished by ion fragment intensity and literature data [37]. Quinic acid esterified with phenylpropanoids in sugarcane has been reported frequently.

## 4. Discussion

Statistical methods, especially based on multivariate analyses, have largely contributed to the meaning of metabolomics data [37,38]. In the present study, we have showed LC-MS metabolomics as a useful tool to comprehend changes during phenological phases of sugarcane subjected to five nematicide controls (two carbamate based, one sulphone compound, a natural product, and one biological composed by *Bacillus* species). 

Supplementation with carbosulfan and *B. subtilis + B. licheniformis* promoted an increase of 9.7% and 6.63% in sugar yield, respectively. Both compounds were previously tested in different varieties of sugarcane and an increment of 10% and 5% was also observed. The authors explained these differences by measuring levels of nematode infestation, which indicate the efficiency of pesticides applied in the studies [39,40]. Carbamates penetrate into the phytoparasite nematode and induce changes in metabolism leading them to death [41]. Meanwhile, *Bacillus* species act through the release of antimicrobial compounds in the soil, it efficiently combat phytopathogenic fungi, bacteria and nematode species [42,43]. 

Despite belong to carbamate class, benfuracarb led to a decrease of 3.96% in sugar production. Likewise benfuracarb, azadirachtin has not demonstrated significant results for increase productivity. Both treatments showed CQA derivatives as discriminant features in PCA analysis. In those groups, CQAs could be related with lower efficiency to reduce nematode population in sugarcane (RB92579). 

CQAs in plants are associated with defense mechanisms, which includes herbivory [44,45]. NMR metabolomics demonstrated the mobilization of the phenolic pathway and the increase of CQAs in sugarcane leaves in response to *Diatraea saccharalis* [46]. The present work indicate a similar response of sugarcane to nematode infestation. In addition, these metabolites are also related to vegetal growth; they play a pivotal role in plant cell wall formation, acting as the building blocks of lignin [47,48]. 

Lignification is also a defense mechanism against herbivory. However, at present it is a hindrance in the obtention of sugarcane byproducts, mainly sugar and bioethanol. It occurs because not only lignin amount but also their composition influencing the content of sucrose and other fermented sugars [49]. Besides, phenolics from alternative pathways, including flavonoids, are also involved in lignification [50]. Our data suggests that differences in CQAs profile, 125 days after planting may led to lower growth and reduced levels of sugar productivity. Literature reports that silencing the expression and inhibition of key enzymes from lignin pathway, during earlier stages of sugarcane development, improves sugar content and lignocellulose saccharification [51,52,53].

This work open fields for novel investigations in agrochemicals, their impact on crops, and the association of CQAs in the production of sugarcane. Supported by proper research, CQAs might become a useful marker to predict levels of infestation by parasites and satisfactory industrial yield.

## 5. Conclusions

The current work verified physiological and metabolic responses of sugarcane (Variety: RB92579) to five different nematicides. Biometric values indicated that benfuracarb and carbosulfan had an influence on production. Chemical profile data revealed differences in phenolic content among the treatments; twenty-seven compounds were identified by LC-MS experiments, including flavonoids and caffeoyl quinic acid (CQAs) derivatives. According to statistical analysis, CQAs compose the main features that differentiate each group. The presence of these compounds in benfuracarb-treated crops suggests an association with higher levels of nematodes infestation; hence, the impact on plant growth and sugar production levels. 

The search for economically viable, less toxic agrochemicals is vital. Nematode control in sugarcane is a challenging task for farmers and companies. In order to ally gains in productivity and reduce environmental damages, understanding metabolic changes as response to integrated strategies of control should be considered in order to pave the way for future management of sugarcane and other crops.

## Figures and Tables

**Table 1:** Biometric characteristics and agro-industrial data from sugarcane collected from Fazenda Santa Emília II.

Agro-Industrial and Biometric Variables									
Fazenda Santa Emília Ii-Field 14									
Products	Plants/Meter	Height (m)	Culm Diameter (mm)			Weight (kg)	Agricultural Productivity (t ha^−1^)	TRS * (kg)	TSH ** (t ha^−1^)
			Base	Quite	Apex				
T1—Control	10.4 ± 0.6	2.4 ± 0.2	25 ± 1.5	22.9 ± 1.2	20.1 ± 1.2	520 ± 61.6	78.7 ± 9.3	156.8 ± 8.3	12.36
T2—*Bacillus subtilis* and *Bacillus licheniformis*	10.5 ± 0.9	2.5 ± 0.2	24.1 ± 1.2	21.9 ± 0.6	19.1 ± 1.1	568 ± 63	86.0 ± 9.5	153.1 ± 5.6	13.18
T3—Benfuracarb	9.9 ± 0.7	2.2 ± 0.2	24.5 ± 1.7	22.4 ± 1.7	19.3 ± 1.5	506 ± 64	76.6 ± 10	154.8 ± 8.7	11.87
T4—Carbosulfan	10.3 ± 0.5	2.4 ± 0.2	24.7 ± 1.6	23.1 ± 1.3	19.9 ± 1.6	574 ± 76.7	86.9 ± 11	155.9 ± 7.1	13.56
T5—Fluensulfone	11.1 ± 0.4	2.4 ± 0.2	25.1 ± 2.2	22.3 ± 1.2	19.2 ± 1.7	544 ± 79	82.4 ± 10	157.1 ± 5.1	12.95
T6—Azadirachtin	10.8 ± 1.1	2.4 ± 0.2	24.3 ± 1.4	22.4 ± 1.4	20 ± 1.6	522 ± 66	79.0 ± 8.9	155.1 ± 7.9	12.27
General Average	10.5	2.4	24.6	22.5	19.6	539	81.66	155.5	12.70

* TRS—Total recovery sugar. ** TSH—Total sugar per hectare.

**Table 2:** Characterization of compounds by HPLC-DAD-ESI-MSn and HR-ESIMS of extract from sugar cane culms (treatments 1, 3, and 4).

Treatment	Peak No.	RT (min)	UV (nm)	*m*/*z* [M−H]^−^	Molecular Formula	Error (ppm)	MS^2^/MS^3^	Annotation	Ref.
1, 3, 4	1	8.1	253	329.0882	C_14_H_17_O_9_	−0.2	MS^2^ [329]: 149(31); 167(100)/MS^3^ [329→167]:122(100)	Vanilic acid glucoside	[27]
1, 3, 4	2	12.3	247	299.0778	C_13_H_15_O_8_	−4.5	MS^2^ [299]: 137(100)/MS^3^ [299→137]: 93(100)	Hydroxybenzoic-4β-glucoside	[27]
1, 3, 4	3	14.0	253	329.0888	C_14_H_17_O_9_	−2.9	MS^2^ [329]: 152(11); 167(100)/MS^3^ [329→167]: 107(18); 123(59); 151(100)	Vanilic acid glucoside-isomer	[28]
1, 3	4	15.0	325	353.0890	C_16_H_17_O_9_	−3.5	MS^2^ [353]: 135(10); 139(38); 191(100)/MS^3^ [353→191]: 85 (55); 127(100); 173(79)	3-caffeoylquinic acid	[29]
1, 3	5	15.6	307	337.1105	C_13_H_21_O_9_	5.0	MS^2^ [337]: 163(100); 191(12)/MS^3^ [337→163]: 119 (100)	Coumaroylquinic acid	[29]
1, 3	6	18.0	326	353.0876	C_16_H_17_O_9_	0.5	MS^2^ [353]: 191(100)/MS^3^ [353→191]: 85(56); 127(100); 173(81)	1-caffeoylquinic acid	[29]
1, 3	7	18.6	323	353.0865	C_16_H_17_O_9_	3.6	MS^2^ [353]: 173(100); 179(56); 191(41)/MS^3^ [353→173]: 71(13); 93(100); 111(58); 155(16)	4-caffeoylquinic acid	[29]
1, 3	8	20.2	323	353.0875	C_16_H_17_O_9_	1.0	MS^2^ [353]: 173(2); 179(3); 191(100)/MS^3^ [353→191]: 85 (71); 127(100); 173(86);	5-caffeoylquinic acid	[29]
1, 3, 4	9	20.9	276	579.1348	C_26_H_28_O_15_	0.4	MS^2^ [579]: 369(36); 399(59); 429(15); 441(21); 459(100); 471(6); 489(25)/MS^3^ [579→459]: 369(100); 399(85); 441(25)	Luteolin-6-C-glucosyl -8-C-arabinosidel	[27]
1, 3, 4	10	21.7	275	579.1351	C_26_H_28_O_15_	0.4	MS^2^ [579]: 369(55); 399(62); 411(5); 429(19); 441(9); 459(35); 471(19); 489(100); 519(18); 561(10)/MS^3^ [579→489]: 369(100); 399(65); 429(6)	Luteolin-6-C-arabinosyl-8-C-glucoside	[27]
1, 3, 4	11	21.8	271	563.1401	C_26_H_28_O_14_	1.2	MS^2^ [563]: 353(100); 365(14); 383(90); 413(17); 425(19); 443(48); 473(63); 503(42); 545(13)/MS^3^ [563→353]: 297(100); 325(99)	Apigenin-6-C-arabinosyl-8-C-glucoside	[27]
1, 3	12	22.1	271	579.1375	C_26_H_28_O_15_	−2.8	MS^2^ [579]: 369(36); 399(37); 429(15); 459(10); 471(11); 489(100); 519(23); 561(10)/MS^3^ [579→489]: 369(100); 399(32); 471(5)	Luteolin-6-C-arabinosyl-8-C-glucoside (isomer)	[27]
1, 3, 4	13	22.1	313	563.1404	C_26_H_28_O_14_	−0.8	MS^2^ [563]: 297(6); 353(97); 365(5); 383(75); 413(11); 425(10); 443(100); 473(63); 503(42); 545(13)/MS^3^ [563→443]: 297(100); 325(99)	Apigenin-6-C-glucosyl-8-C-arabinoside	[27]
1, 3, 4	14	22.7	340	447.0921	C_21_H_19_O_11_	2.7	MS^2^ [477]: 327(99); 357(100); 429(27)/MS^3^ [477→357]: 285(27); 297(67); 311(23); 327(13) 339(100)	Luteolin-8-C-glucoside (isoorientin)	[27]
1, 3, 4	15	22.7	272	593.1514	C_27_H_30_O_15_	0.4	MS^2^ [593]: 309(30); 327(33); 339(19); 357(30); 429(52); 473(100) 575(6)/MS^3^ [593→473]: 297(37); 327(100)	Apigenin-6.8-C-diglucoside	[30]
1, 3, 4	16	23.6	275	431.0971	C_21_H_20_O_10_	2.9	MS^2^ [431]: 283(7); 311(100); 341(9)/MS^3^ [431→311]: 283(100)	Apigenin-8-C-glucoside (vitexin)	[30]
1, 3, 4	17	23.6	328	563.1432	C_26_H_28_O_14_	−4.6	MS^2^ [563]: 353(97); 383(58); 413(13); 443(100); 473(73); 485(7) 503(6); 515(10); 545(9)/MS^3^ [563→443]: 353(100); 383(15)	Apigenin-6-C-glucosylarabinoside	[31]
1, 3, 4	18	24.2	337	447.0913	C_21_H_20_O_11_	4.3	MS^2^ [477]: 285(100)/MS^3^ [477→285]: 151(13); 175(52); 217(60); 257(21); 267(17)	Luteolin-7-O-glucoside	[32]
1, 3	19	24.3	328	515.1186	C_25_H_24_O_12_	1.8	MS^2^ [515]: 353(100)/MS^3^ [515→353]: 135(8); 179(39); 191(100)	3-5-dicaffeoylquinic acid	[27]
1, 3, 4	20	24.8	270	431.0976	C_21_H_20_O_10_	1.7	MS^2^ [431]: 311(100); 341(37); 413(8)/MS^3^ [431→311]: 283(100)	Apigenin-6-C-glucoside (Isovitexin)	[30]
1, 3, 4	21	24.2	270	577.1556	C_27_H_30_O_14_	1.1	MS^2^ [577]: 293(99); 311(7); 323(11); 335(5); 341(9); 413(100); 457(13)/MS^3^ [577→413]: 283 (100)	Apigenin-6-C-glucosylrhamnoside	[27]
1, 3, 4	22	25.4	271	461.1088	C_22_H_22_O_11_	0.4	MS^2^ [461]: 285(6); 341(100); 371(24);/MS^3^ [461→431]: 297(100); 313(18)	Diosmetin-6-C-glucoside	[27]
1, 3	23	25.3	285	515.1182	C_25_H_24_O_12_	2.5	MS^2^ [515]: 353(100)/MS^3^ [515→353]: 135(6); 173(100); 179(65); 191(31)	3-4-dicaffeoylquinic acid	[27]
1, 3, 4	24	26.4	270	461.1087	C_22_H_22_O_11_	0.4	MS^2^ [461]: 283(20); 299(100); 341(6)/MS^3^ [461→299]: 283(100)	Diosmetin-8-C-glucoside	[27]
1, 3, 4	25	27.1	270	651.1523	C_36_H_28_O_12_	-2.3	MS^2^ [651]: 271(16); 299(21); 313(32); 329(100)/MS^3^ [651→329]: 299(7); 314(100)	Tricin-7-O-a-L-rhamnosyl-glucuronide	[27]
1, 3, 4	26	27.8	270	585.0544	C_23_H_22_O_16_S	2.0	MS^2^ [585]: 255(100)/MS^3^ [585→255]: 97(11); 133(5); 157(10); 167(12); 175(100); 193(96); 211(16); 237(9)	Tricin-7-O-glucuronide-sulfate	[27]
1, 3, 4	27	29.2	271	577.1554	C_27_H_30_O_14_	1.5	MS^2^ [577]: 413(18); 431(100)/MS^3^ [577→431]: 369(63); 413(100)	Apigenin -8-C-glucosylrhamnoside	[27]

## Data Availability

The authors confirm that the data supporting the findings of this study are available within the article and its supplementary materials. Additional information of this study are available from the corresponding author MTS on request.

## Supplementary Materials

The following supporting information can be downloaded at: https://www.mdpi.com/article/10.3390/life13041034/s1, Figure S1. Randomized block design with five replications. Table S1. Results of Total Recoverable Sugar (TRS) analyses for each treatment (T). Figure S2. (A) Base peak chromatogram (BPC) in negative ion mode of T1 (collection 4). (B). 2D Expansion showing the main ions present in sugarcane stem extract between 4–7.0 min by HPLC-DAD-ESI-MS^n^. Figure S3. Expansion of the Base peak chromatogram (BPC) in negative ion mode between 4–7.0 min of T1 to T6. (collection 4) by HPLC-DAD-ESI-MS^n.^ Figure S4. Graph of (left) PCA scores and (right) loadings (PC1 vs. PC2) for treatments 2 (red) and 4 (blue) analyzed by mass spectrometry. Figure S5. Graph of (left) PCA scores and (right) loadings (PC1 vs. PC2) for treatments 3 (red) and 6 (green) and 1 (blue) analyzed by mass spectrometry. Figure S6. Graph of (left) PCA scores and (right) loadings (PC1 vs. PC2) for treatments 3 (blue) and 6 (red) analyzed by mass spectrometry. Figure S7. HRMS spectra of the main features annotated and listed in Figure 5. All the figures contain the molecular formula and retention time of each feature.
