# Supplementary material for: Mass Spectrometry-Based Investigation of Sugarcane Exposed to Five Different Pesticides
Source: Life (Basel). 2023 Apr 17;13(4):1034. doi: 10.3390/life13041034 (PMC10145413; doi:10.3390/life13041034)
Supplement: Supplementary file 1 [file life-13-01034-s001.zip › life-2244773-supplementary.pdf]

Supporting Information

Figure S1. Randomized block design with five replications.

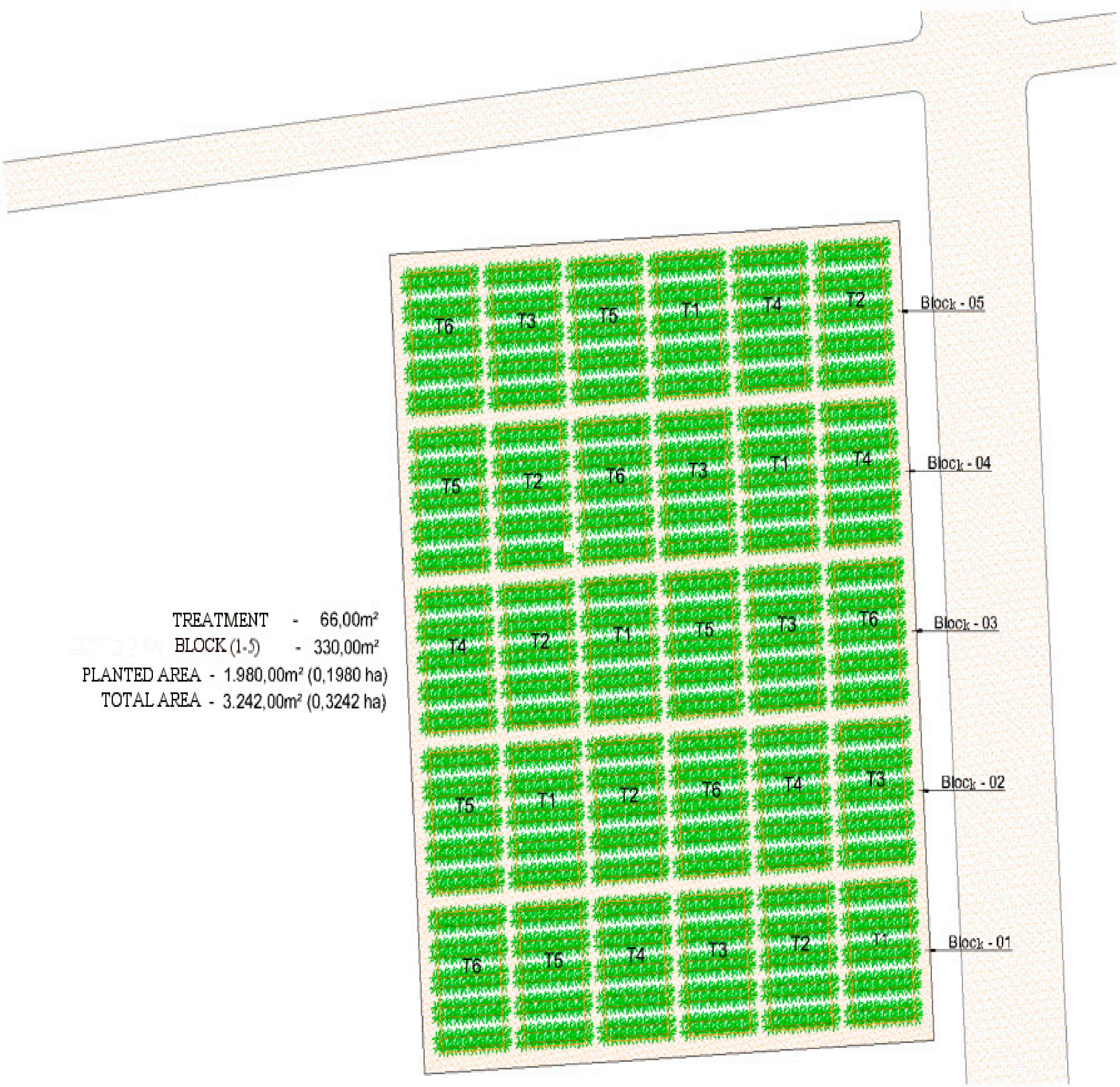

Table S1. Results of Total Recoverable Sugar (TRS) analyses for each treatment (T).

	T1	TRS	T2	TRS	T3	TRS	T4	TRS	T5	TRS	T6	TRS
28/11/2019	**B1**	150.13	**B1**	157.91	**B5**	143.9	**B1**	158.83	**B2**	157.66	**B1**	163.21
28/11/2019	**B2**	161.88	**B2**	144.09	**B1**	153.6	**B2**	152.54	**B3**	156.85	**B2**	172.37
28/11/2019	**B3**	160.16	**B3**	154.91	**B2**	171.9	**B3**	147.24	**B4**	152.88	**B3**	144.02
28/11/2019	**B4**	165.75	**B4**	160.42	**B3**	152.16	**B4**	160.44	**B5**	159.1	**B4**	154.79
28/11/2019	**B5**	156.95	**B5**	149.57	**B4**	155.85	**B5**	148.48	**B1**	151.28	**B5**	160.72
09/01/2020	**B1**	148.58	**B1**	155.1	**B1**	154.33	**B1**	162.08	**B1**	154.14	**B1**	154.67
09/01/2020	**B2**	156.64	**B2**	147.74	**B2**	138.13	**B2**	152.91	**B2**	159.58	**B2**	160.95
09/01/2020	**B3**	149.54	**B3**	156.3	**B3**	168.95	**B3**	158.2	**B3**	155.93	**B3**	154.93
09/01/2020	**B4**	165	**B4**	153.38	**B4**	140.85	**B4**	159.34	**B4**	158.5	**B4**	155.33
09/01/2020	**B5**	148.75	**B5**	158.6	**B5**	151.79	**B5**	152.05	**B5**	155.6	**B5**	150.85
05/11/2020	**B2**	157.49	**B5**	163.12	**B4**	164.29	**B4**	154.37	**B4**	163.9	**B5**	156.21
05/11/2020	**B5**	151.89	**B3**	148.86	**B5**	152.44	**B5**	151.70	**B1**	159.03	**B3**	148.92
05/11/2020	**B3**	170.10	**B4**	141.31	**B3**	158.62	**B3**	177.18	**B2**	161.5	**B4**	136.68
05/11/2020	**B1**	140.41	**B1**	151.22	**B1**	158.70	**B1**	151.86	**B3**	166.32	**B1**	156.32
05/11/2020	**B4**	169.67	**B2**	155.19	**B2**	157.15	**B2**	151.54	**B5**	144.6	**B2**	156.72
**Average**	-	**156.86**	-	**153.18**	-	**154.8**	-	**155.9**	-	**157.12**	-	**155.11**
**Standard deviation**	-	**8.37**	-	**5.66**	-	**8.78**	-	**7.11**	-	**5.1**	-	**7.97**

***B - Block**

Figure S2. (**A**) Base peak chromatogram (BPC) in negative ion mode of T1 (collection 4). (**B**). 2D Expansion showing the main ions present in sugarcane stem extract between 4–7.0 min by HPLC-DAD-ESI-MS^n.

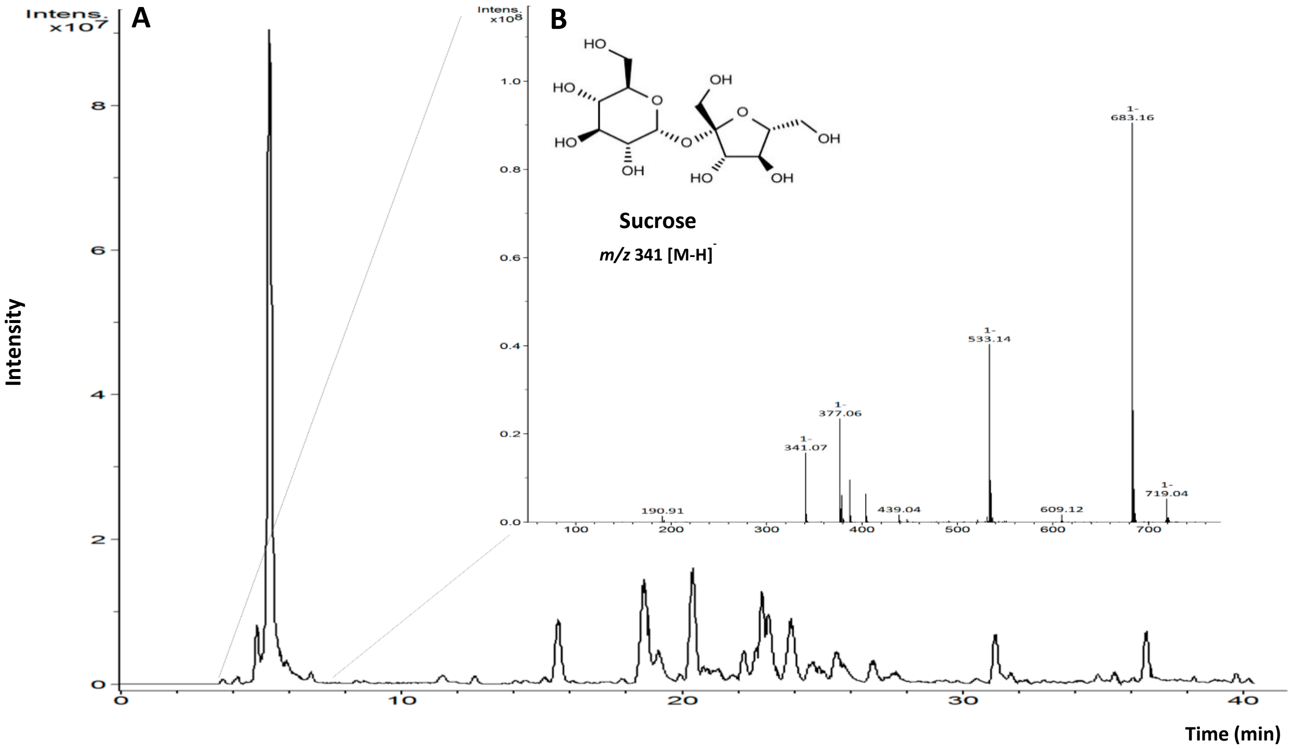

Figure S3. Expansion of the Base peak chromatogram (BPC) in negative ion mode between 4–7.0 min of T1 to T6. (collection 4) by HPLC-DAD-ESI-MS^n.

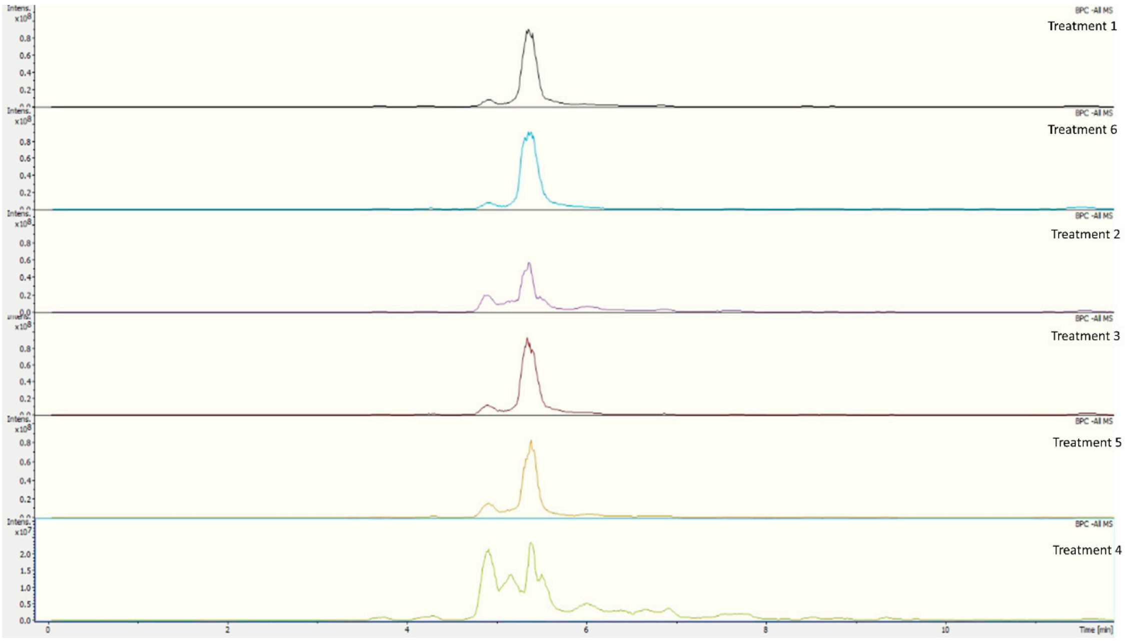

Figure S4. Graph of PCA scores (**left**) and loadings plots (PC1 vs. PC2) (**right**) for treatments 2 (red) and 4 (blue) analyzed by mass spectrometry.

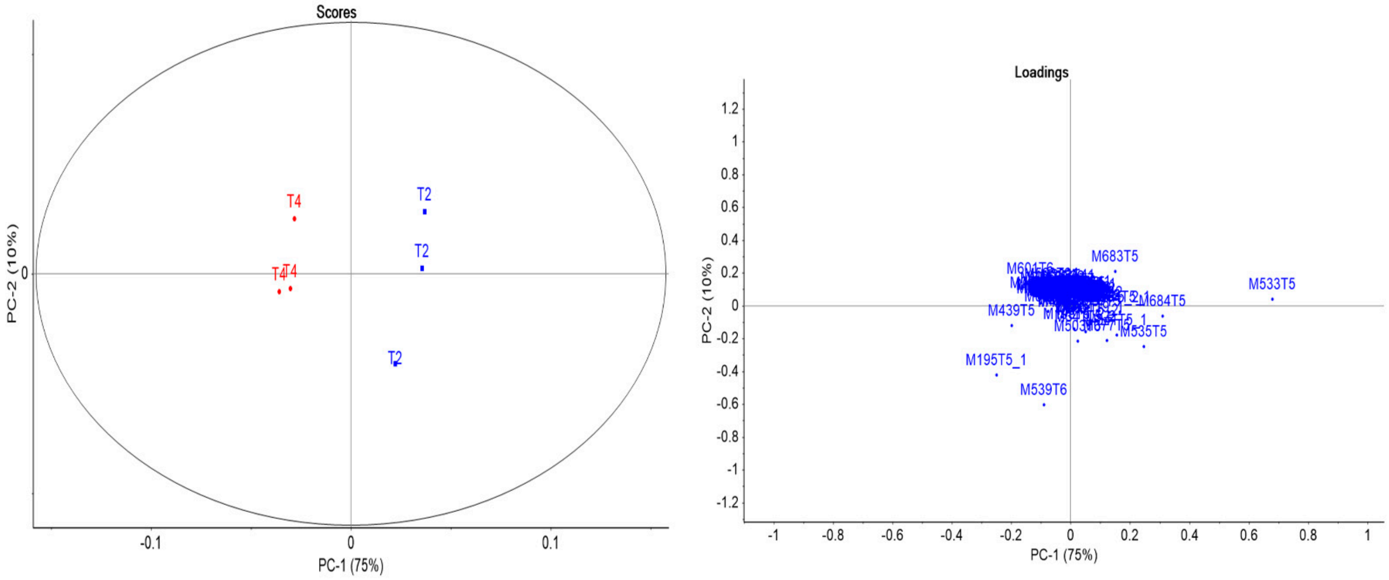

Figure S5 Graph of PCA scores (**left**) and loadings plots (PC1 vs. PC2) (**right**) for treatments 3 (red) and 6 (green) and 1 (blue) analyzed by mass spectrometry.

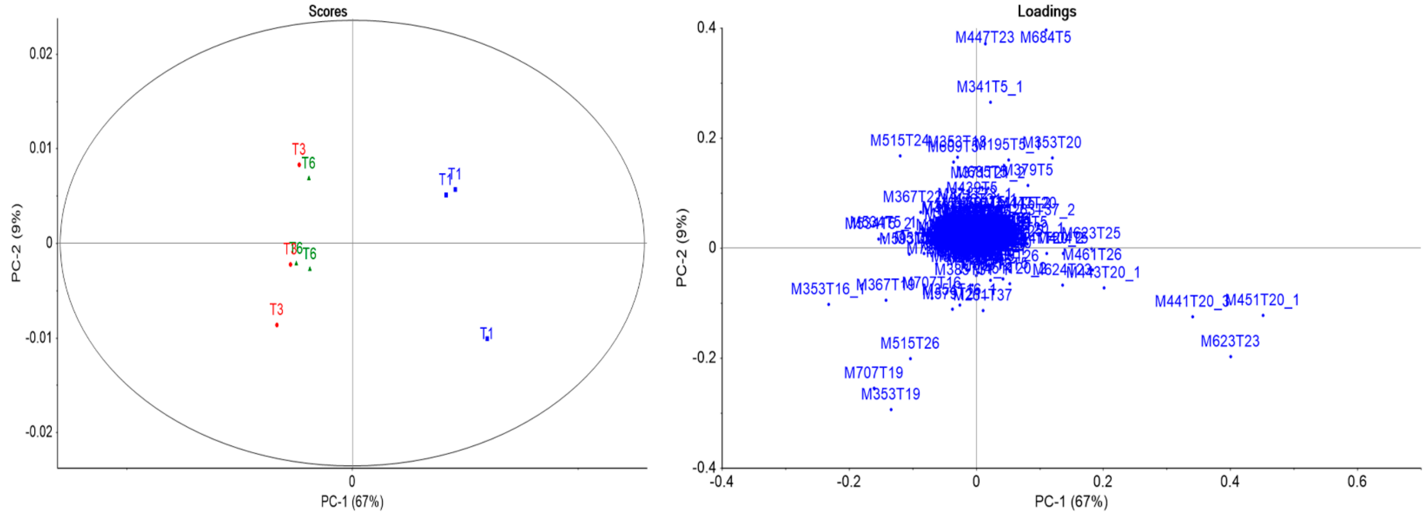

Figure S6. Graph of PCA scores (**left**) and loadings plots (PC1 vs. PC2) (**right**) for treatments 3 (blue) and 6 (red) analyzed by mass spectrometry.

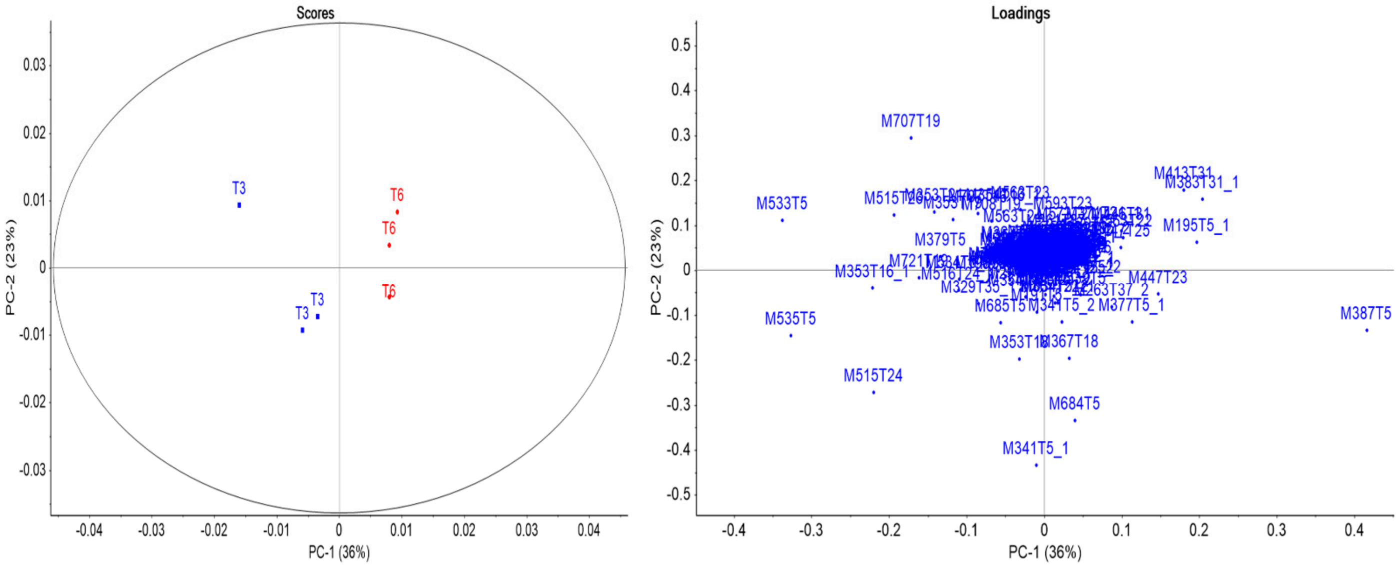

Figure S7. HRMS spectra of the main features annotated and listed in table 3. All the figures contain the molecular formula and retention time of each feature.

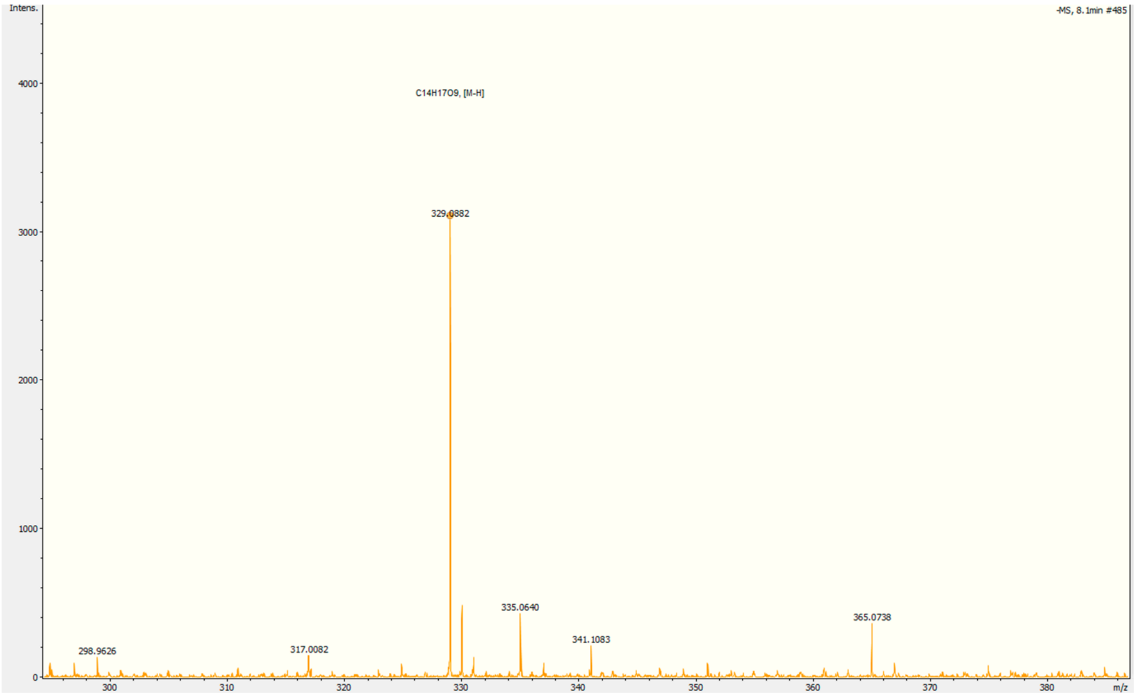

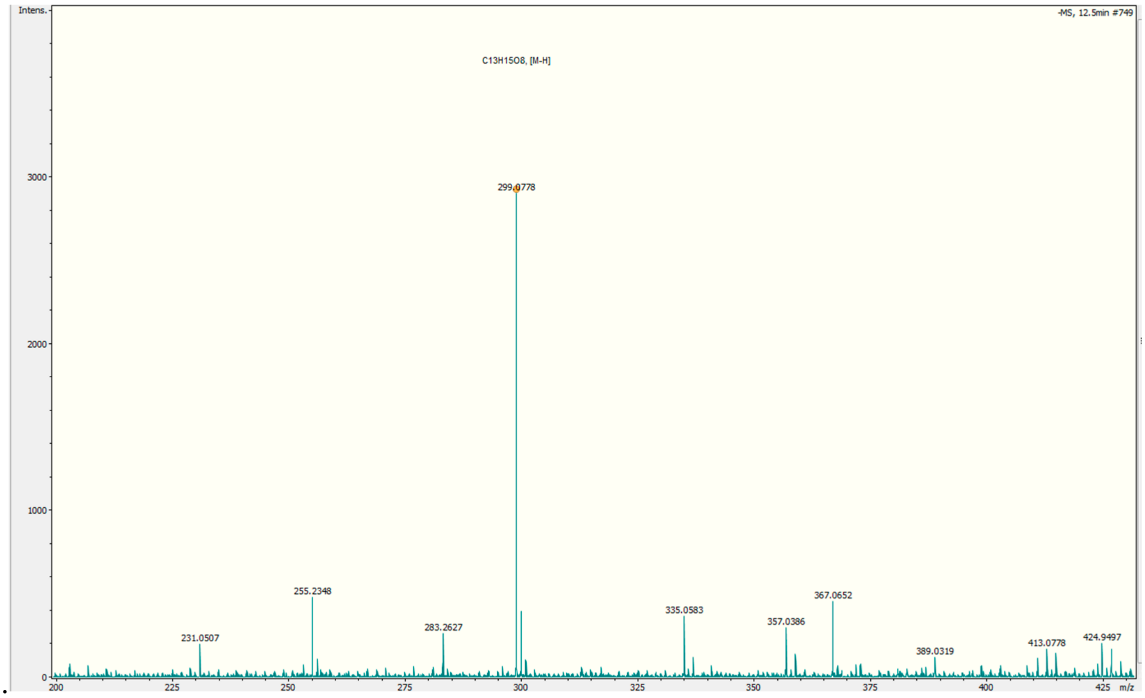
Intens.
-MS, 12.5min #749
C13H15O8, [M-H]
3000
2000
1000
0
299.0778
255.2348
231.0507
283.2627
335.0583
357.0386
367.0652
389.0319
413.0778
424.9497
200
225
250
275
300
325
350
375
400
425
m/z

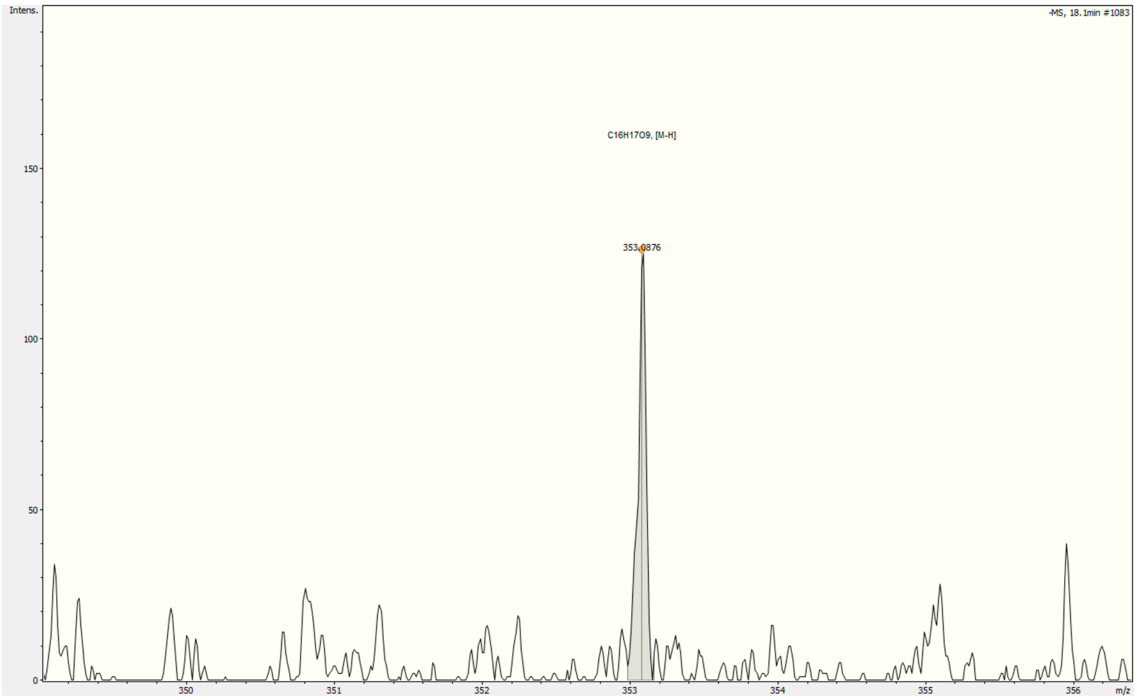
Intens.
-MS, 18.1min #1083
C16H17O9, [M-H]
150
100
50
0
353.0876
350
351
352
353
354
355
356
m/z

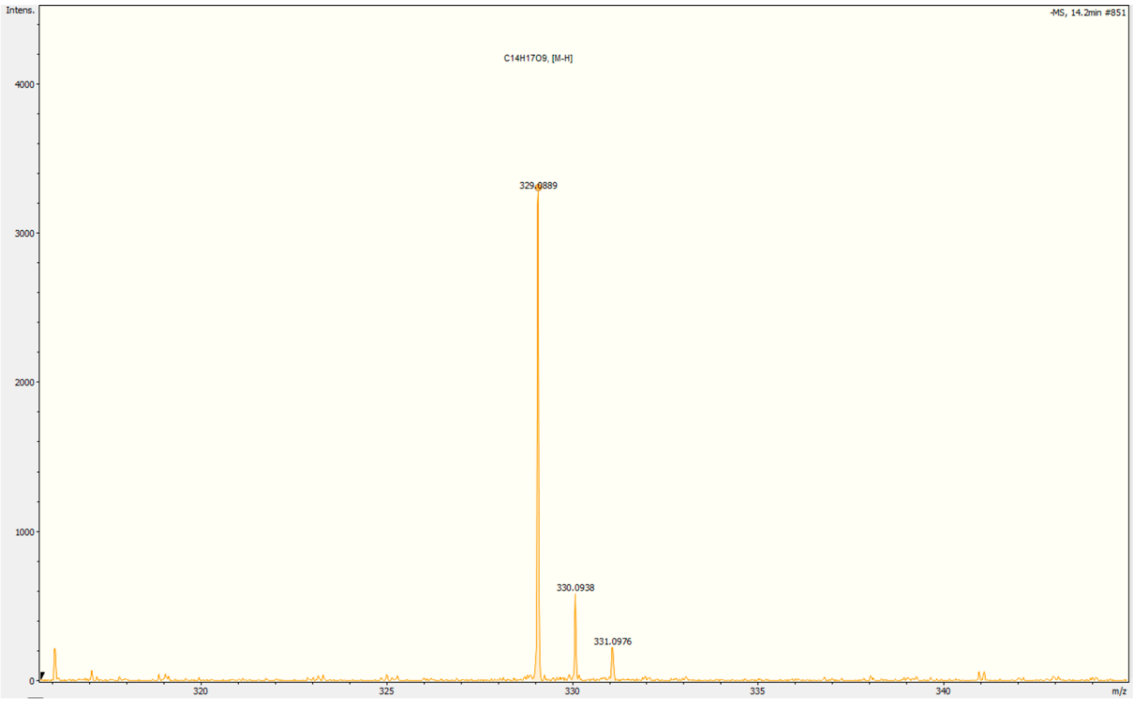
Intens.
-MS, 14.2min #851
C14H17O9, [M-H]
329.0889
330.0938
331.0976
4000
3000
2000
1000
0
320
325
330
335
340
m/z

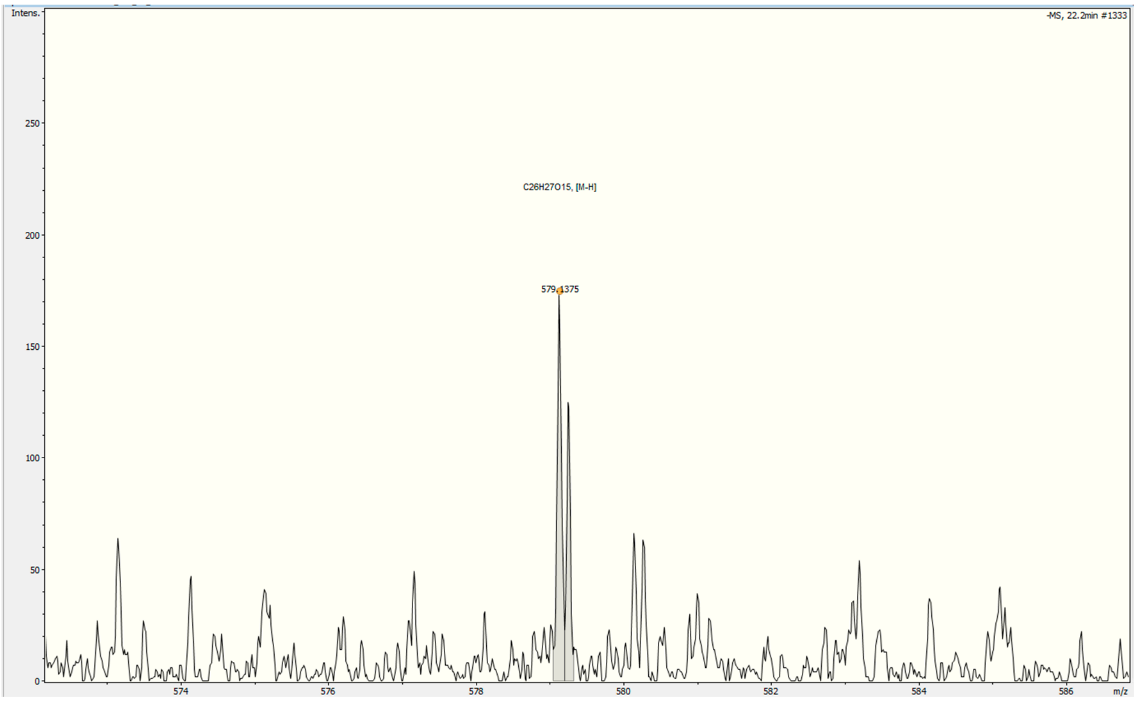
Intens.
-MS, 22.2min #1333
C26H27O15, [M-H]
579.1375
250
200
150
100
50
0
574
576
578
580
582
584
586
m/z

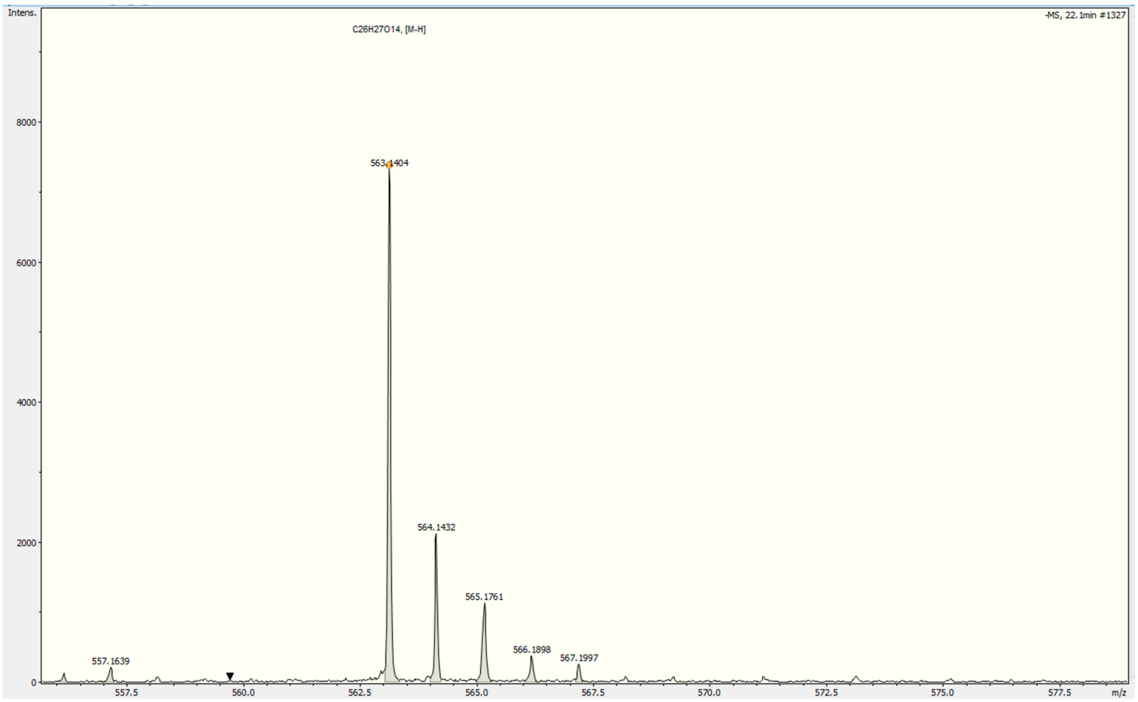
Intens.
-MS, 22.1min #1327
C26H27O14, [M-H]
563.1404
564.1432
565.1761
566.1898
567.1997
557.1639
m/z

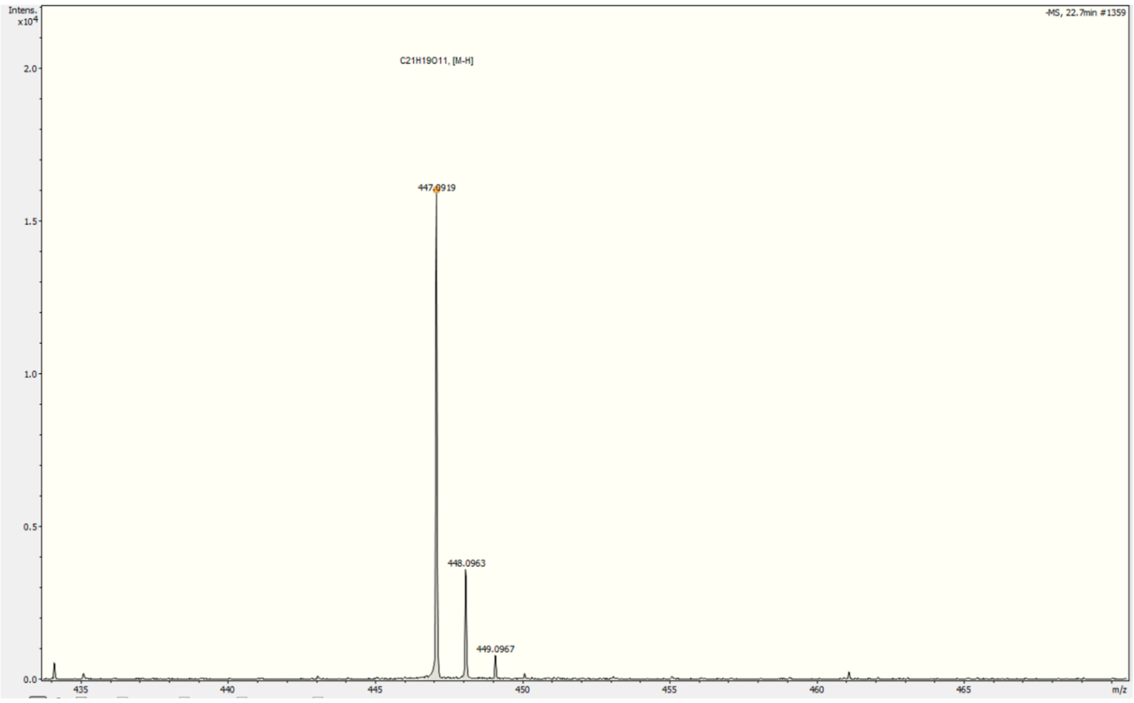
Intens.
x10^4
-MS, 22.7min #1359
C21H19O11, [M-H]
447.0919
448.0963
449.0967
m/z

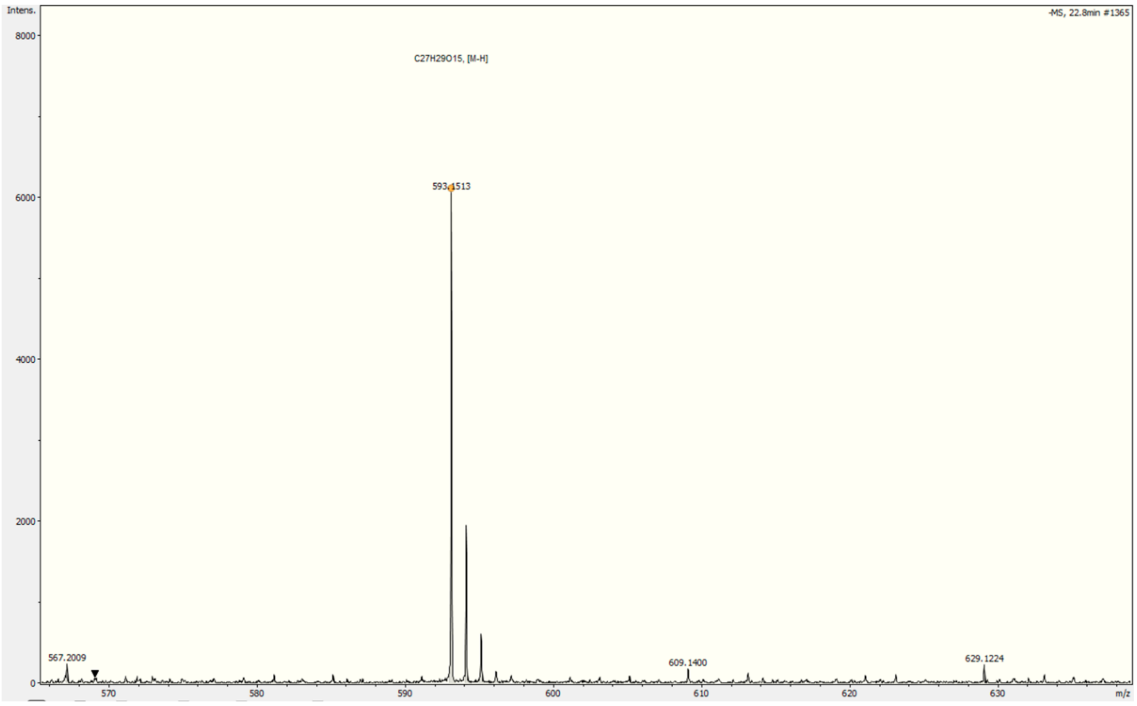
Intens.
-MS, 22.8min #1365
C27H29O15, [M-H]
593.1513
567.2009
609.1400
629.1224
m/z

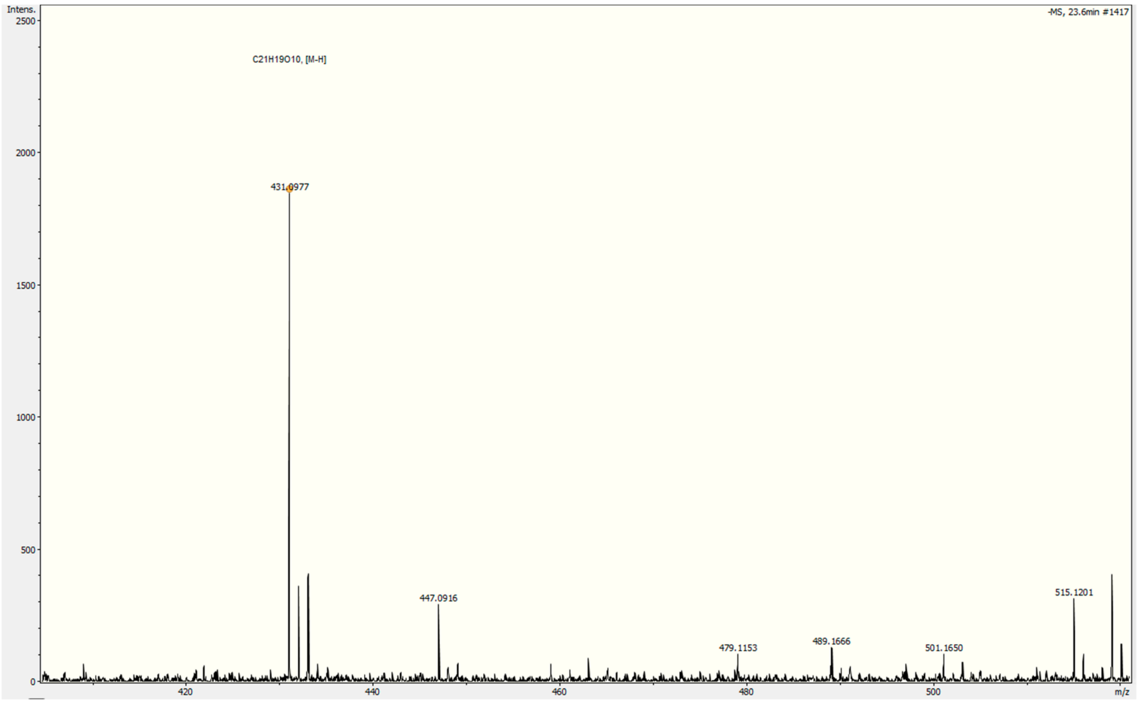
Intens.
-MS, 23.6min #1417
C21H19O10, [M-H]
431.0977
447.0916
479.1153
489.1666
501.1650
515.1201
m/z

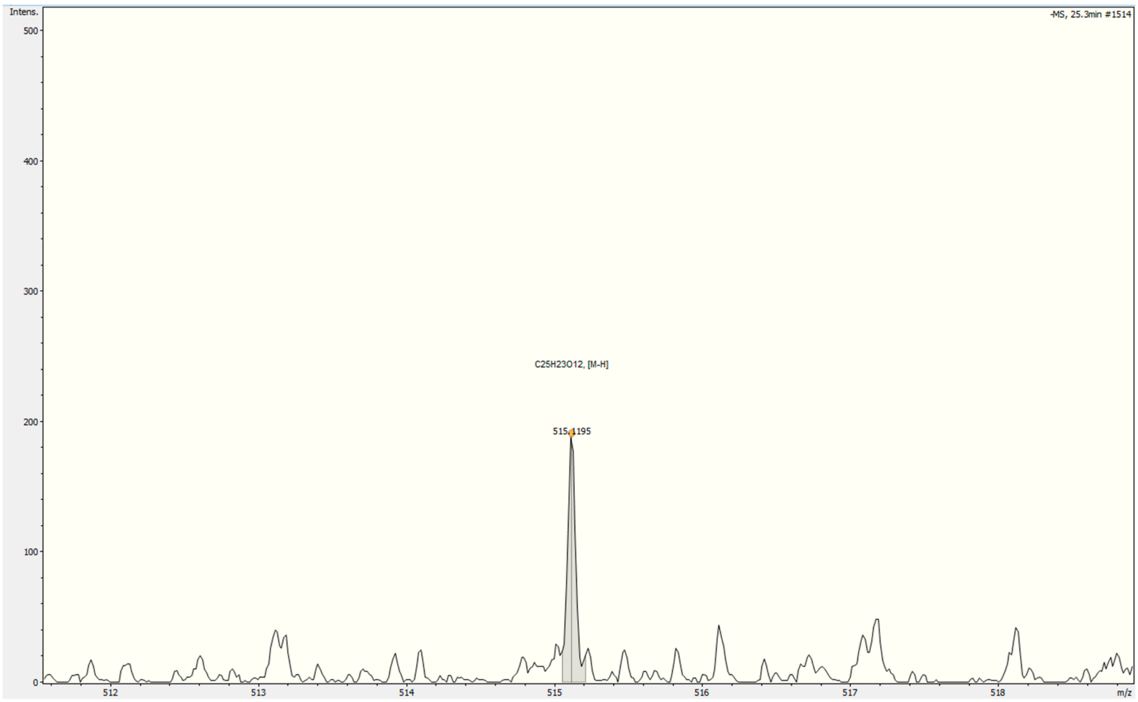
Intens.
-MS, 25.3min #1514
C25H23O12, [M-H]
515.1195
m/z

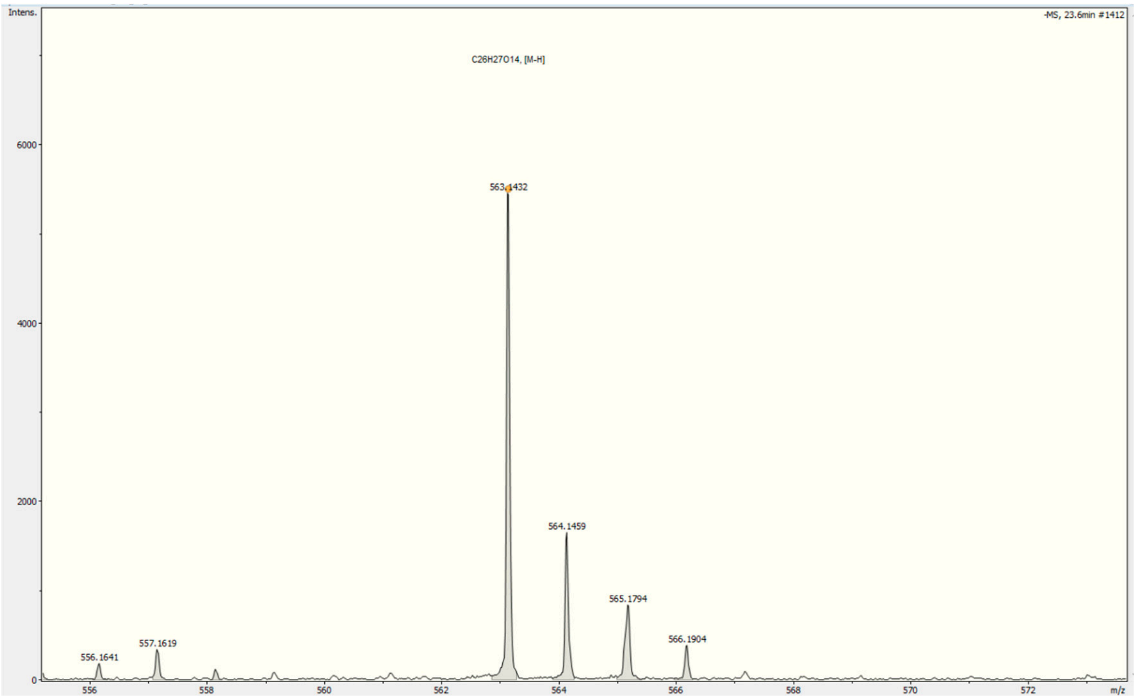
Intens.
-MS, 23.6min #1412
C26H27O14, [M-H]
563.1432
564.1459
565.1794
566.1904
556.1641
557.1619
m/z

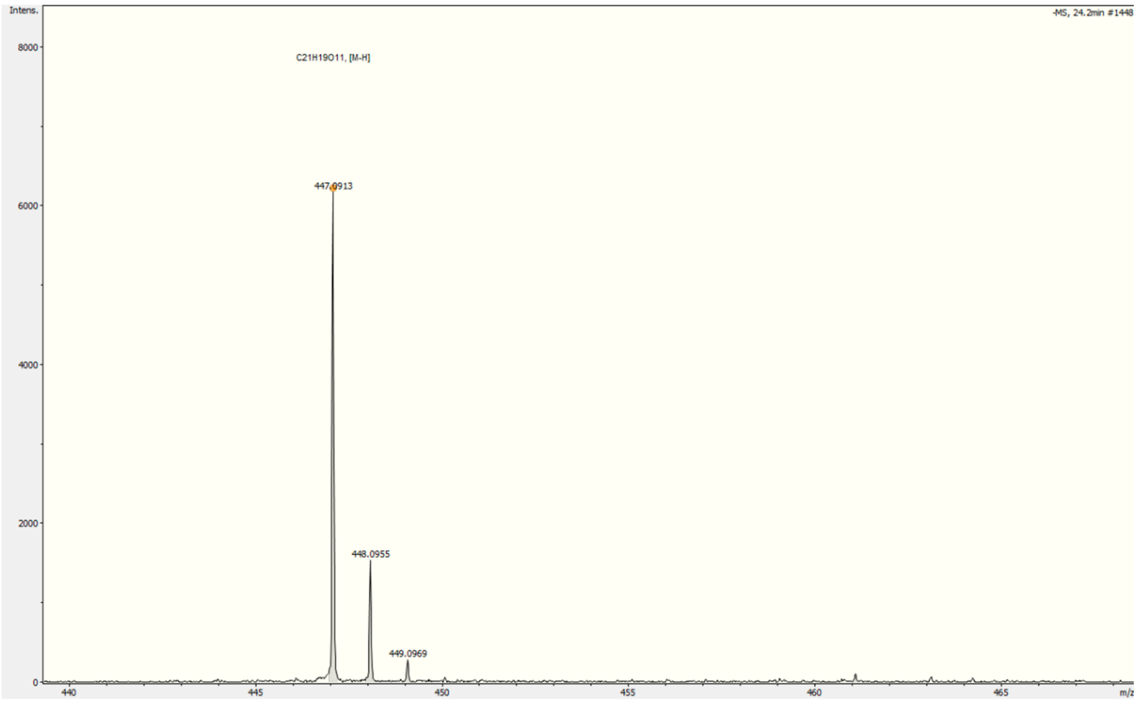
Intens.
-MS, 24.2min #1448
C21H19O11, [M-H]
447.0913
448.0955
449.0969
m/z

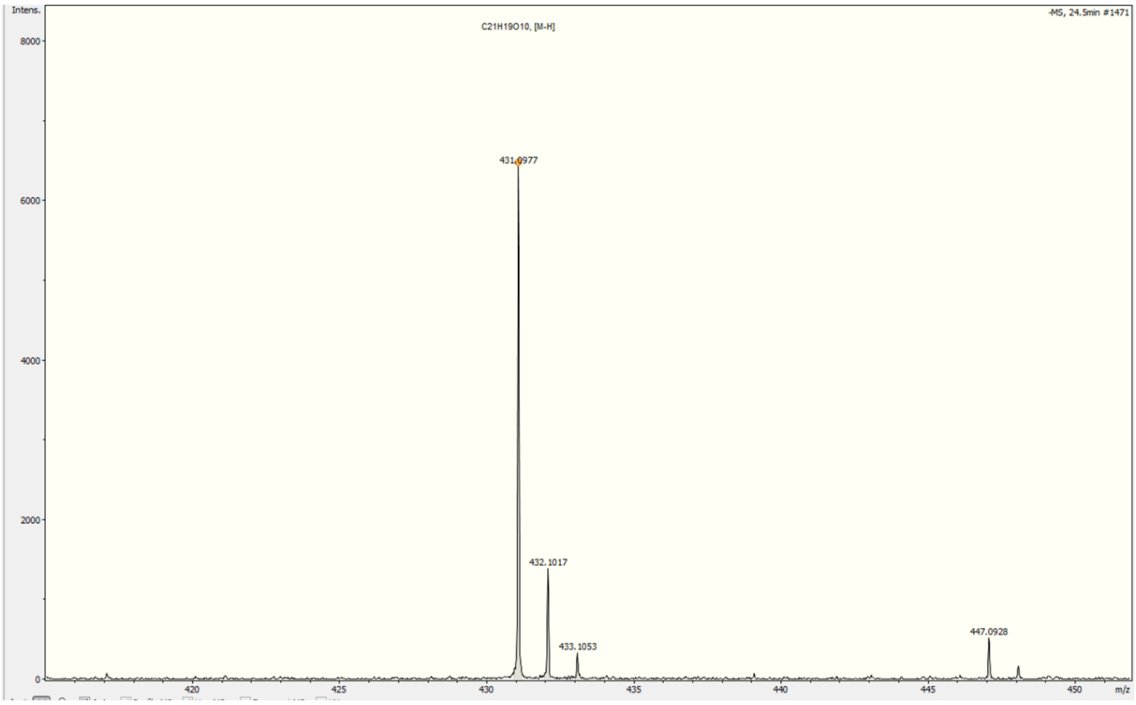
Intens.
-MS, 24.5min #1471
C21H19O10, [M-H]
431.0977
432.1017
433.1053
447.0928
m/z

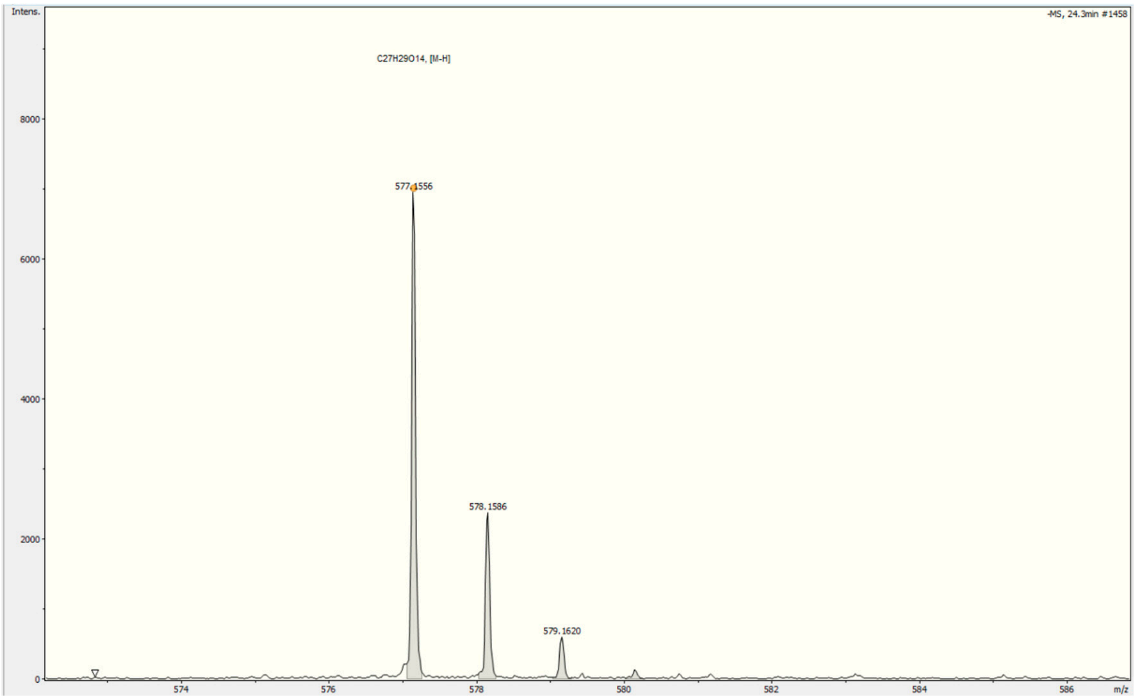
Intens.
-MS, 24.3min #1458
C27H29O14, [M-H]
577.1556
578.1586
579.1620
m/z

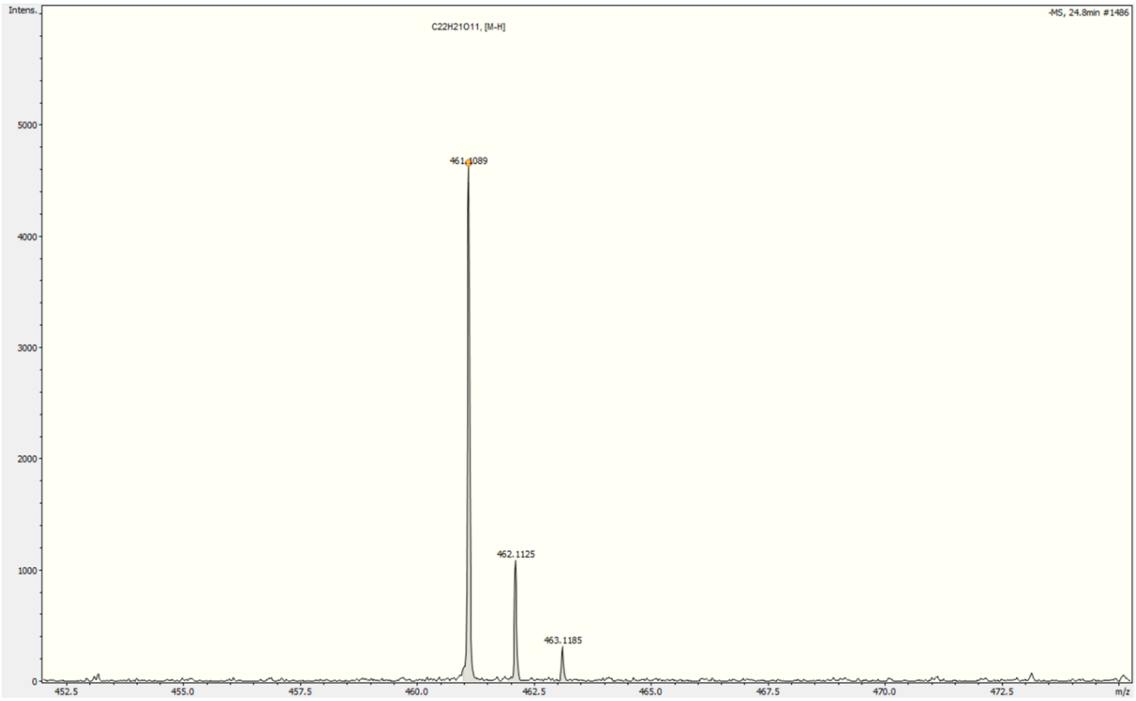
Intens.
-MS, 24.8min #1486
C22H21O11, [M-H]
461.1089
462.1125
463.1185
m/z

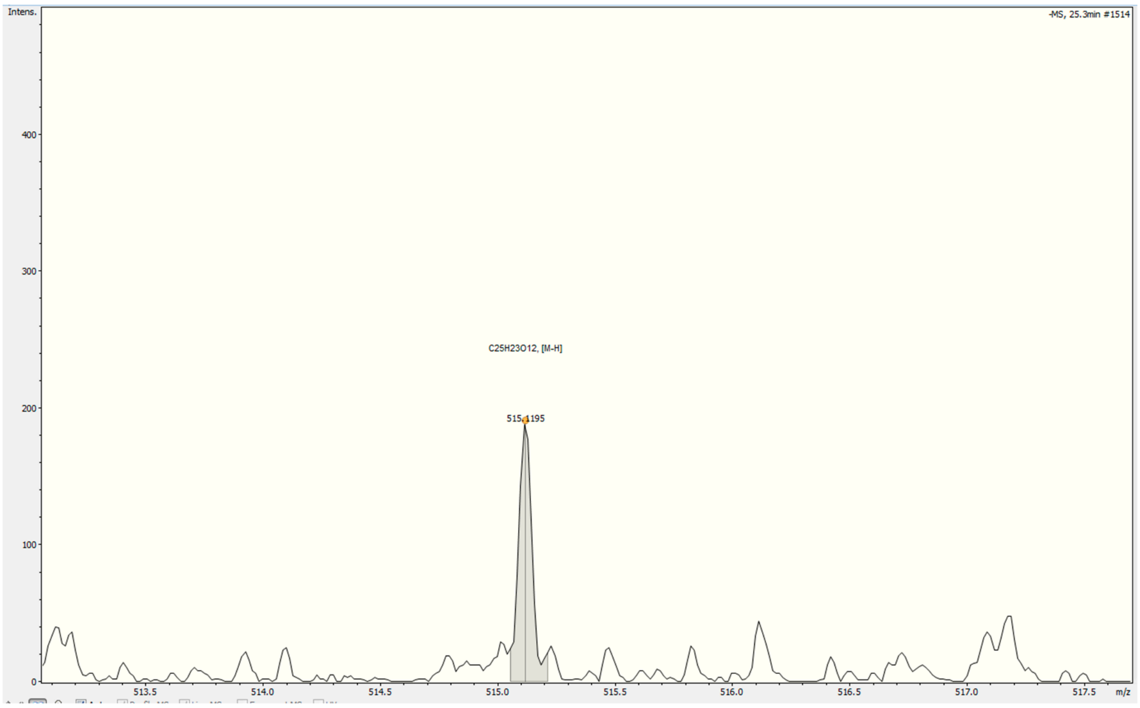
Intens.
-MS, 25.3min #1514
C25H23O12, [M-H]
515.1195
m/z

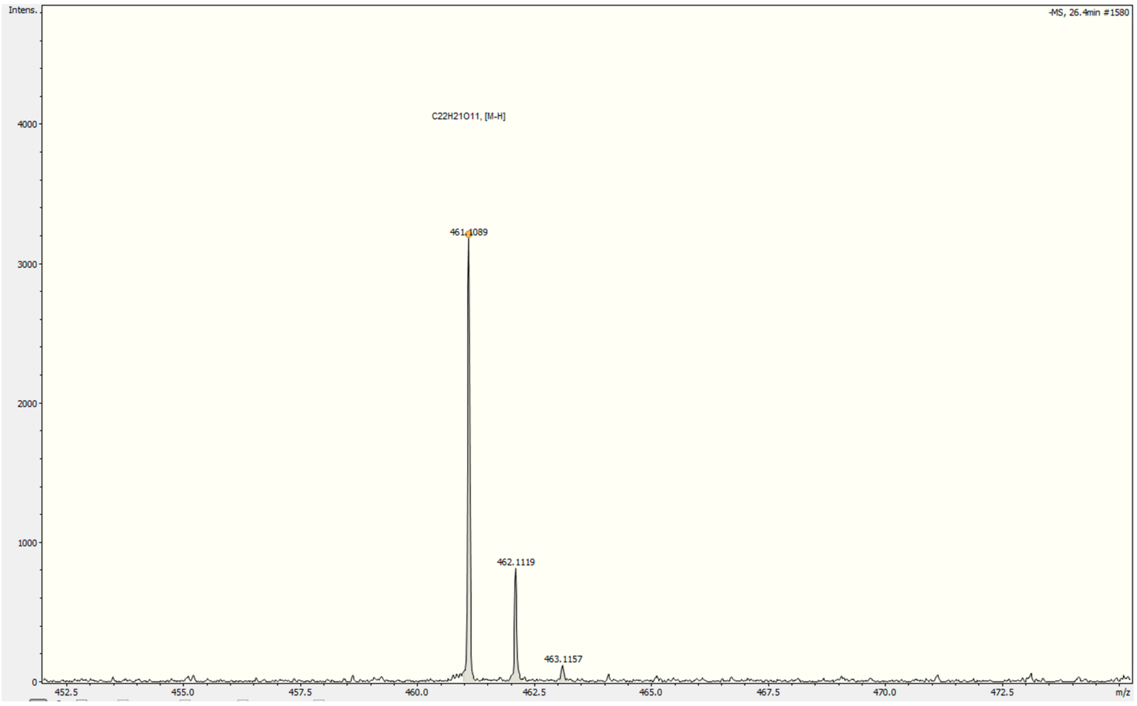
Intens.
-MS, 26.4min #1580
C22H21O11, [M-H]
461.1089
462.1119
463.1157
m/z

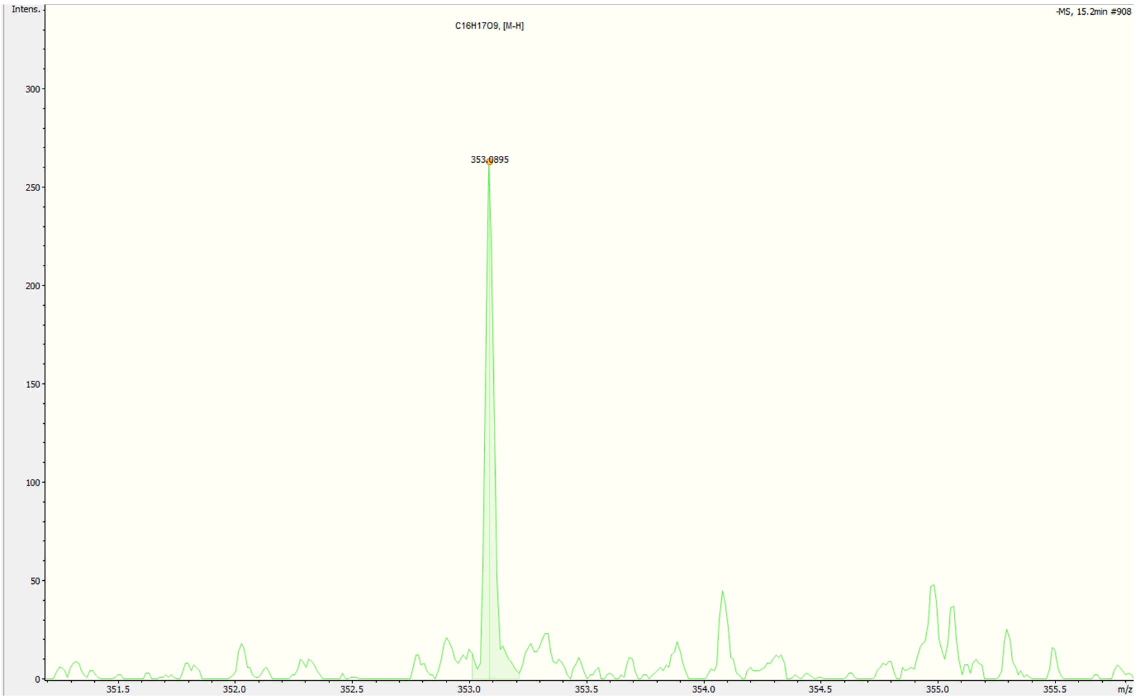

Intens.
C16H17O9, [M-H]
-MS, 15.2min #908
353.0895
m/z

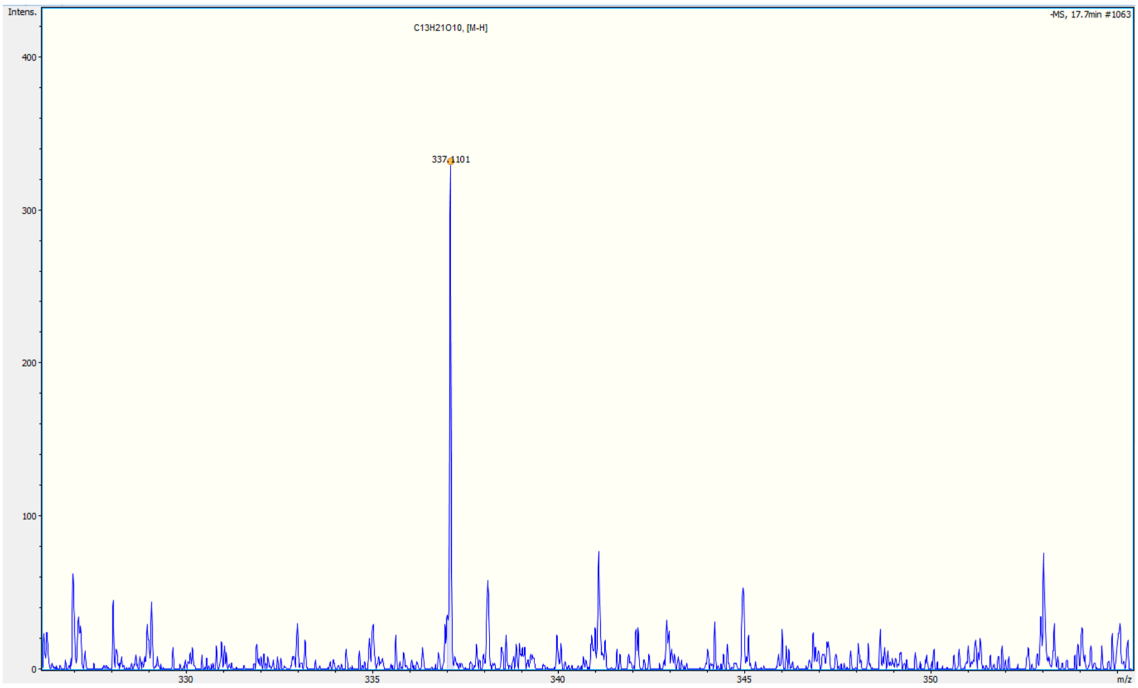

Intens.
C13H21O10, [M-H]
-MS, 17.7min #1063
337.1101
m/z

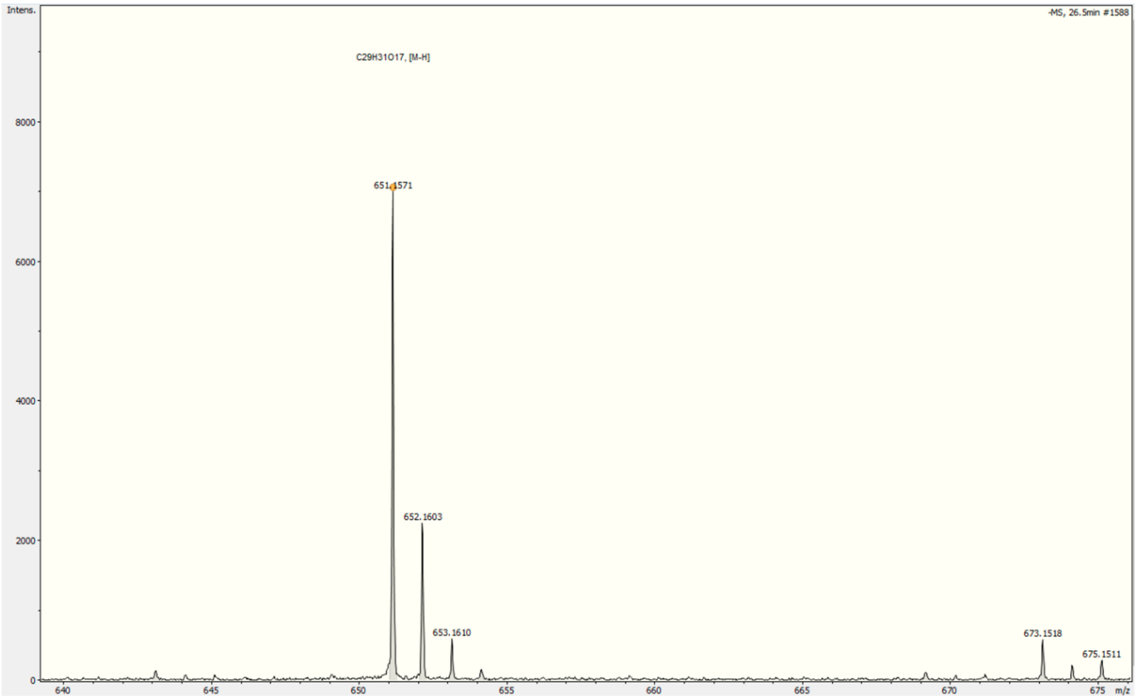
Intens.
-MS, 26.5min #1588
C29H31O17, [M-H]
651.1571
652.1603
653.1610
673.1518
675.1511
m/z

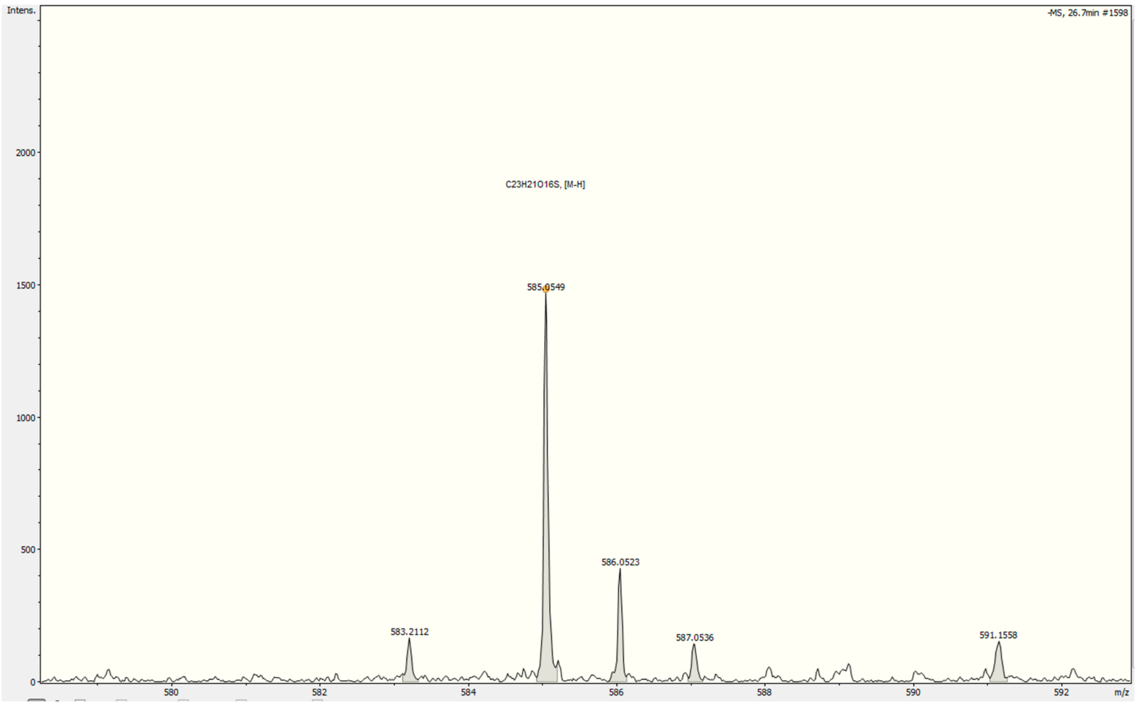
Intens.
-MS, 26.7min #1598
C23H21O16S, [M-H]
585.0549
586.0523
583.2112
587.0536
591.1558
m/z

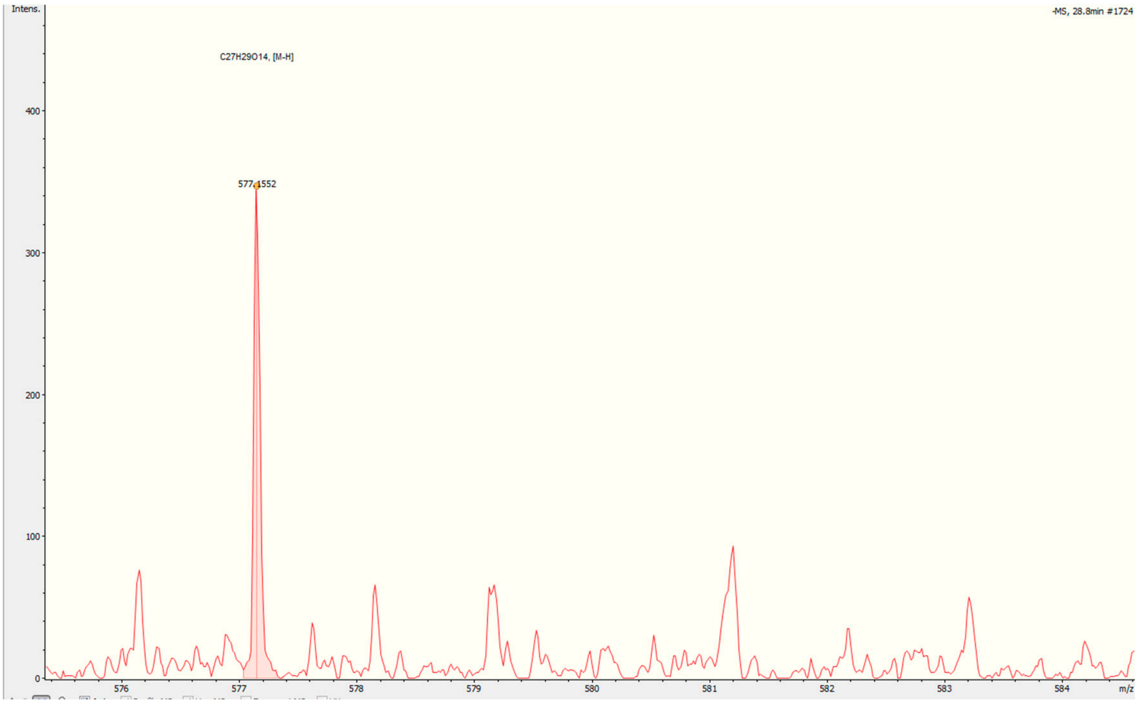
Intens.
-MS, 28.8min #1724
C27H29O14, [M-H]
577.1552
400
300
200
100
0
576
577
578
579
580
581
582
583
584
m/z
